# Bidirectional Allosteric Coupling between PIP_2_ Binding and the Pore of the Oncochannel TRPV6

**DOI:** 10.3390/ijms25010618

**Published:** 2024-01-03

**Authors:** Christina Humer, Tamara Radiskovic, Kata Horváti, Sonja Lindinger, Klaus Groschner, Christoph Romanin, Carmen Höglinger

**Affiliations:** 1 Institute of Biophysics, Johannes Kepler University Linz, 4020 Linz, Austria; christina.humer_1@jku.at (C.H.); tamara.radiskovic@jku.at (T.R.); christoph.romanin@jku.at (C.R.); 2 Institute of Materials and Environmental Chemistry, Research Centre for Natural Sciences, 1117 Budapest, Hungary; khorvati@elte.hu; 3 Gottfried Schatz Research Center, Division of Biophysics, Medical University of Graz, 8010 Graz, Austria; klaus.groschner@medunigraz.at

**Keywords:** TRPV6, calcium, PIP_2_, cis-22a, inhibition, lipid

## Abstract

The epithelial ion channel TRPV6 plays a pivotal role in calcium homeostasis. Channel function is intricately regulated at different stages, involving the lipid phosphatidylinositol-4,5-bisphosphate (PIP_2_). Given that dysregulation of TRPV6 is associated with various diseases, including different types of cancer, there is a compelling need for its pharmacological targeting. Structural studies provide insights on how TRPV6 is affected by different inhibitors, with some binding to sites else occupied by lipids. These include the small molecule cis-22a, which, however, also binds to and thereby blocks the pore. By combining calcium imaging, electrophysiology and optogenetics, we identified residues within the pore and the lipid binding site that are relevant for regulation by cis-22a and PIP_2_ in a bidirectional manner. Yet, mutation of the cytosolic pore exit reduced inhibition by cis-22a but preserved sensitivity to PIP_2_ depletion. Our data underscore allosteric communication between the lipid binding site and the pore and vice versa for most sites along the pore.

## 1. Introduction

Beyond merely acting as structural components of semipermeable boundaries, membrane lipids fulfill multiple, essential functions within a cell. Depending on the specific lipid species, they may constitute modulators of various types of proteins and act as signaling molecules. As such, lipids are involved in diverse cellular processes like migration, membrane trafficking, cytoskeletal dynamics, and, among others, transmembrane passage of metabolites and ions [1,2,3]. A highly relevant lipid in these regards is phosphatidylinositol-4,5-bisphosphate (PIP_2_). It is derived upon consecutive phosphorylation of the inositol head group of phosphatidylinositol (PI) at positions 4 and 5, respectively, at the plasma membrane. Despite representing one of the most abundant phosphorylated PI derivatives in the plasma membrane, PIP_2_, which is specifically enriched in the layer facing the cytosol, constitutes only approximately 1–2 mol% of all plasma membrane lipids [2,4].

PIP_2_ synthesis by phosphatidylinositol kinases is dynamic, as is its depletion via phosphatases and by the enzyme phospholipase C (PLC). This allows for dynamic regulation of PIP_2_-dependent proteins, including ion channels. Depending on the specific channel, PIP_2_ can promote or inhibit channel activity. Moreover, sensitivity to PIP_2_ can altogether restrict channel activity to specific cellular compartments, as reported for mucolipin-type transient receptor potential (TRPML) channels, which remain inactive in the plasma membrane due to PIP_2_ but are activated by phosphatidylinositol 3,5-bisphosphate of intracellular membranes [2,4,5,6,7]. Instead, PIP_2_ is important to keep the voltage-dependent potassium channels KCNQ1-5 [8,9,10], different inward rectifier K^+^ channels [11,12,13,14], or the melastatin-type TRP channel TRPM5 [15] active. Moreover, PIP_2_ serves as a positive regulator of the voltage-dependent Ca^2+^ channel CaV2.2 [16], as well as of the vanilloid-type TRPs TRPV5 [17,18] and TRPV6 [19,20].

TRPV5 and TRPV6 channels show the highest Ca^2+^ selectivity within the large TRP channel family, serving as key components of renal Ca^2+^ reabsorption and uptake of dietary Ca^2+^, respectively [21,22,23,24,25,26,27,28,29]. Apart from mediating membrane entry of Ca^2+^ into epithelial cells of the kidney and the intestine [26,27,28], TRPV6, which is less restricted in its expression pattern than TRPV5, is, for instance, also present in the placenta, where it is important for maternal–fetal Ca^2+^ transport [30,31,32]. Also, TRPV6 protein is detectable in exocrine organs like sweat, salivary, and mammary glands as well as the prostate or pancreas. Within these tissues, malfunctioning of the channel or changes in its expression levels are strongly linked to diseases, including cancer [24,33,34]. TRPV6 has, for instance, been found to be overexpressed in leukemia and malignancies of thyroid, colon, pancreas, prostate, ovaries, or breast cancer cells. In many cases, TRPV6 overexpression shows a strong linkage to tumor aggressiveness, cell migration and viability [24,35,36,37,38,39]. Therefore, reducing TRPV6-based Ca^2+^ influx is a highly interesting therapeutic strategy and inhibition of TRPV6 has already been examined in clinical trials [38,39,40,41,42,43].

TRPV6 proteins contain six transmembrane domains (TM1-TM6), as well as large N- and C-terminal portions within the cytosol. Homomeric channels are assembled from six TRPV6 proteins, with TM5 and TM6, together with a membrane-entrant loop–helix–loop section (pore loop) intercalating these, composing the pore domain [25,44,45,46]. The channel periphery is formed by TM1-TM4, which associate with the pore domain in a domain-swapped arrangement, i.e., residing in closer proximity to TM5-TM6 of a neighboring TRPV6 protomer rather than the one of the same subunit [45,46]. The interfaces between the individual subunits of the channel serve as association sites for lipids [46,47,48,49,50]. For channels including those composed of closely related TRPV5 proteins, cryogenic electron microscopy (cryo-EM) structural investigations succeeded in resolving presumptive PIP_2_ binding sites [8,10,51,52,53,54]. Yet, for TRPV6, none of the four different resolved non-protein densities matches PIP_2_ in shape [46,47,48,49,55]. Still, the presence of positively charged residues within the binding pocket intercalated between TM3, TM4, and the TM4-TM5 linker of one subunit and TM5 as well as TM6 of the neighboring protomer would predestine this site, termed as lipid binding site 2 (LBS-2, see Supplementary Figure S1A), for interactions with PIP_2_ [46]. Interestingly, the size of the LBS-2 density was found to be larger in the open rather than the closed state of the channel, pointing to the engagement of LBS-2 by an activating lipid like PIP_2_ [46].

Moreover, LBS-2 serves as one of two binding sites of the (4-phenylcyclohexyl)piperazine derivative (PCHPD) cis-22a. The pore of the channel itself composes the other, presumptive major binding site, with associated cis-22a blocking the ion permeation pathway. In addition to physical occlusion, the pore conformation changes into a non-conductive state upon cis-22a binding [47]. The role of associated cis-22a within the membrane-embedded part of the channel for inhibition was thus far difficult to reconcile. Yet there are data indicating that binding to LBS-2 imposes allosteric effects on the pore [47]. Also, the association of an activating lipid with the TRPV6 channel is very likely to induce conformational alterations of the pore so that activating effects can eventuate [45,46,56]. An influence of LBS-2 on the pore is consistently supported by structural comparison of the open and closed state, with the former depicting a larger size of the non-protein density in LBS-2 and considerable conformational changes within the pore at the section below the selectivity filter. These include an α-to-π helical transition in TM6, rotation and bending towards the channel periphery [46].

The presumptive crosstalk between LBS-2 and the pore, indicated by previous electrophysiological data on sensitivity to cis-22a and structurally yet not functionally upon comparing closed and open state channels, prompted us to investigate allosteric regulation of TRPV6 in more detail. Thus, we introduced mutations within these two functionally important regions of the channel, i.e., into LBS-2 and the pore, and analyzed effects on sensitivity to PIP_2_ depletion and cis-22a treatments. Thereby, the analyzed LBS-2 and most yet not all pore mutants which are less responsive to cis-22a treatments interestingly also show a lower inhibition by PIP_2_ depletion. Moreover, we demonstrate that thus far rarely used methods to interfere in protein–PIP_2_ interactions, peptide-based PIP_2_ sequestration, as well as optogenetic PIP_2_ depletion are suitable for studying TRPV6 regulation by PIP_2_. Altogether, our findings thereby provide further proof that PIP_2_ is an important regulator of TRPV6 activity and reveal that allosteric communication between LBS-2 and the pore is at most positions bidirectional rather than monodirectional.

## 2. Results

### 2.1. Electrostatic Sequestration of PIP_2_ Reduces TRPV6 Currents

**Figure 1:**
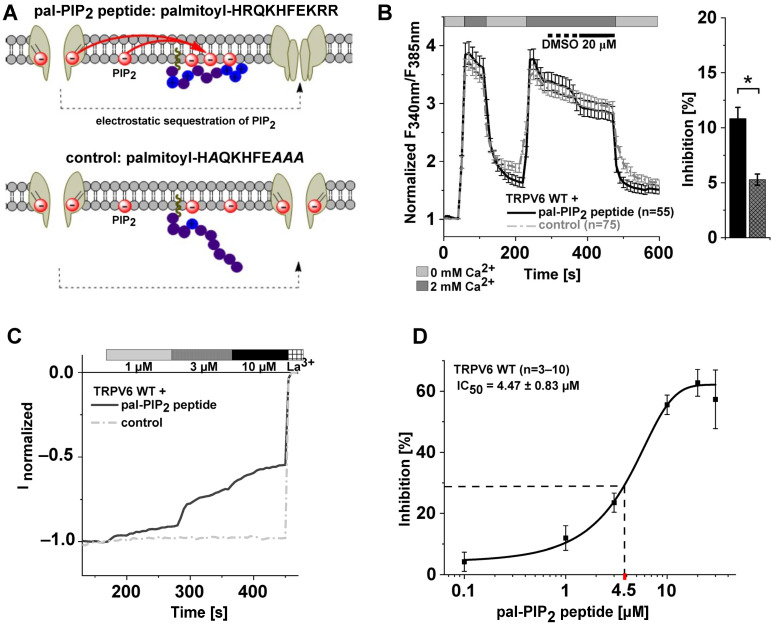
TRPV6 activity is sensitive to pal-PIP_2_ peptide administration. (**A**) Scheme of electrostatic PIP_2_ sequestration by positive charges of the pal-PIP_2_ peptide (**upper panel**). The consequent lower amount of free PIP_2_ within the membrane leads, depending on the respective interaction strength, to dissociation of the lipid from other proteins, given constant equilibration between free PIP_2_ and protein-bound PIP_2_. This is impeded by substituting positively charged residues by alanine in the palmitoylated control peptide (**lower panel**). (**B**) (**left**): Normalized Fura-2 measurements (mean ± SEM) on HEK293 cells overexpressing TRPV6 WT treated either with 20 μM pal-PIP_2_ peptide (black solid line) or the control peptide (grey dashed line). Before peptide addition, accordingly diluted DMSO was perfused as solvent control, as indicated. (**right**): Corresponding mean reduction (±SEM) in signal intensity after peptide administration; the asterisk (*) indicates statistical significance (*p* < 0.05) between treatment with the pal-PIP_2_ peptide (black) and the control peptide (grey). (**C**) Representative normalized whole-cell current of TRPV6 WT-transfected HEK293 cells obtained during perfusion with the indicated dose of either the pal-PIP_2_ peptide (black, solid) or the control peptide (grey, dashed). At the end of the measurements, currents were blocked by 1 mM La^3+^, as indicated. (**D**) Dose–response curve of TRPV6 WT inhibition by the pal-PIP_2_ peptide using a Hill fit and the calculated IC_50_ value (means ± SEM; Hill coefficient: 1.95).

TRPV6 channels show constitutive activity and are negatively regulated by PLC or phosphatase-mediated PIP_2_ depletion [19,20,57]. Upon investigating PIP_2_ regulation of TRPV6, we sought to circumvent the generation of reaction products having functions in signaling inherent to standard approaches, including enzyme-based conversion or inhibition of re-synthesis [54,58,59]. Therefore, we tested whether TRPV6 activity is affected by lowering the effective concentration of PIP_2_ within the membrane by electrostatic sequestration. HEK293 cells overexpressing TRPV6 were thus treated with a positively charged decapeptide derived from the PIP_2_ binding domain of a voltage-gated potassium channel. Such treatment already proved to be able to modulate potassium currents and serotonin transporter activity [60,61]. For membrane passage and anchorage within the cytosolic leaflet, a palmitoyl group is linked to the N-terminus of the peptide [60], in our case, via a carboxamide bond, and the C-terminus was present as carboxamide as well instead of a free carboxyl moiety. This PIP_2_-binding pal-peptide is subsequently referred to as pal-PIP_2_ peptide (Figure 1A, upper panel). For control measurements, an analogously designed palmitoylated peptide was implemented having four of the basic residues of the pal-PIP_2_ peptide replaced by alanine (Figure 1A, lower panel, Table 1), as established by Buchmayer et al. (2013) [61] and herein termed as control for simplicity.

Initial Fura-2 measurements revealed a decrease in the cytosolic Ca^2+^ level by approximately 11% when treating TRPV6-transfected HEK293 cells with 20 μM of the pal-PIP_2_ peptide. This decrease was significantly stronger than the slow, continuous reduction observed when applying the control peptide at the same concentration (Figure 1B), an effect likely attributable to ongoing channel inactivation. For a closer characterization, we monitored the effects of different concentrations of the pal-PIP_2_ peptide ranging from 0.1 μM to 30 μM in whole-cell patch clamp recordings. Further increases in the concentration up to 100 μM led to a rapid membrane rupture or leaky measurements, supposedly due to concomitant increases in the DMSO concentration, and were thus not available for further analyses.

As shown in Figure 1C,D (Supplementary Figure S1B–D), TRPV6-overexpressing HEK293 cells showed typical inward rectifying currents with a highly positive reversal potential (E_rev_ > +50 mV), consistent with the considerable Ca^2+^ selectivity of TRPV6. Current density was reduced in a dose-dependent manner when applying the PIP_2_-binding pal-peptide but not the control, supporting that the reduction in Ca^2+^ currents is indeed due to interference in the regulation of TRPV6 by PIP_2_. As exemplarily shown for 10 μM pal-PIP_2_ peptide, Ca^2+^ selectivity remained unchanged upon adding the peptide (Supplementary Figure S1D). Interestingly, even upon applying relatively high doses of the pal-PIP_2_ peptide, maximum inhibition of TRPV6 currents plateaued at about 60%, as observed from a peptide concentration of 20 μM on (Figure 1D). Given this limit in the overall extent, half-maximum inhibition implies a reduction in currents by approximately 30% (Figure 1D), which was achieved at the pal-PIP_2_ peptide concentration (IC_50_) of 4.47 μM, according to the Hill fit of the dose–response analysis (Hill coefficient: 1.95; Figure 1D).

Of note, in all our analysis, levels of inhibition were considerably higher when derived from electrophysiological measurements compared to Ca^2+^ imaging experiments, what is interestingly a common observation when comparing inhibitory levels yielded from different methods [47,49,62]. In this regard, it needs to be emphasized that the patch clamp technique directly monitors Ca^2+^ influx via TRPV6 channels, whereas in Ca^2+^ imaging experiments, resultant changes in intracellular Ca^2+^ levels are yielded from a fluorescent read-out, which are impacted by various cellular processes, as well as dependent on the experimental conditions.

### 2.2. A Mutation-Based Increase in PIP_2_ Affinity Counteracts Inhibition by the Pal-PIP_2_ Peptide

**Figure 2:**
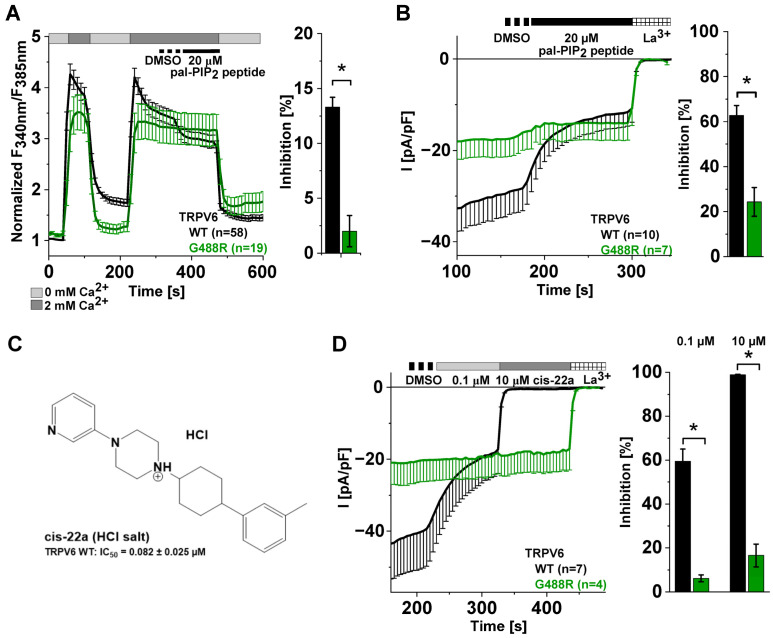
A mutation-based increase in PIP_2_ affinity reduces TRPV6 sensitivity to PIP_2_ depletion and lowers inhibition by cis-22a. (**A**) (**left**): Normalized Fura-2 measurements (mean ± SEM) on HEK293 cells expressing TRPV6 WT (black) or TRPV6 G488R (green) treated with 20 μM pal-PIP_2_ peptide subsequent to the solvent control (DMSO). (**right**): Mean inhibition (± SEM) derived from the measurements on TRPV6 WT (black) and TRPV6 G488R (green) shown on the left. (**B**) (**left**): Averaged whole-cell current densities (−SEM) of HEK293 cells transfected with TRPV6 WT (black) or TRPV6 G488R (green). Cells were successively perfused with DMSO, 20 μM pal-PIP_2_ peptide, and 1 mM La^3+^, as indicated. (**right**): Mean inhibition (±SEM) derived from the measurements shown on the left. (**C**) Chemical structure of the TRPV6 inhibitor cis-22a and the previously determined IC_50_ value [47]. (**D**) (**left**): Mean whole-cell current densities (−SEM) recorded from HEK293 cells expressing TRPV6 WT (black) or TRPV6 G488R (green). Throughout the measurements, cells were kept in 10 mM Ca^2+^ solution, containing the consecutively added DMSO, cis-22a (0.1 and 10 μM), and 1 mM La^3+^. (**right**): Block diagram of the respective extents of inhibition by cis-22a relative to the La^3+^ block. In all bar charts, asterisks (*) indicate statistical significance between TRPV6 WT and the mutant (*p* < 0.05).

Supposing that the reduction in TRPV6 channel activity observed upon applying the pal-PIP_2_ peptide was due to a competitive scavenging of PIP_2_ from the plasma membrane, we wondered whether a mutation-based increase in the affinity of the channel for PIP_2_ might counteract inhibition. According to extensive characterization by Velisetty et al. (2016) [59], channels carrying a glycine (G)-to-arginine (R) substitution in the TM4-TM5 loop (TRPV6 G488R) fulfill the criterium of stronger PIP_2_ binding. Within the concerning study, TRPV6 G488R currents were, among others, shown to be largely resistant to enzyme-based PIP_2_ depletion, predestining this mutant as a control for our analysis. Consistently, acute application of 20 μM pal-PIP_2_ peptide during Fura-2 measurements reduced intracellular Ca^2+^ levels of TRPV6 G488R expressing HEK293 cells by only ~2% (Figure 2A). This level is within the range determined for TRPV6 WT upon application of the control peptide (Figure 1B) rather than the pal-PIP_2_ peptide, the latter of which led to a significantly higher reduction of about 13% in the case of TRPV6 WT-transfected cells (Figure 2A). In patch clamp experiments, the apparent inhibition reached about 24% for TRPV6 G488R and 63% for TRPV6 WT upon applying 20 μM pal-PIP_2_ peptide, respectively (Figure 2B). When repeating electrophysiological measurements with half the dose of the pal-PIP_2_ peptide, inhibition of the TRPV6 G488R variant receded to a value of ~4%, whereas TRPV6 WT currents were still strongly inhibited by about 56% (Supplementary Figure S1E).

As mentioned before, the G488R mutation resides within the segment linking the fourth and fifth transmembrane domain of TRPV6. Importantly, different examples exist throughout the entire TRP superfamily, highlighting the importance of the corresponding protein section for a proper channel function, for instance, by serving as a binding site for different agonists or antagonists [63]. In the case of TRPV6, cryo-EM data indicate that linker residues contribute to LBS-2; thus, they are apparently involved in lipidic regulation [46] and part of one of the two binding sites of the small molecule inhibitor cis-22a [47] (Figure 2C). In the latter regard, we have shown in one of our recent publications that the TRPV6 channel becomes largely insensitive to the mentioned inhibitor upon inserting the G488R mutation into the TM4-TM5 linker [62]. Here, we again observed that TRPV6 G488R currents are not inhibited by the application of 0.1 µM cis-22a (Figure 2D), which equals approximately the IC_50_ of 82 nM of this blocker [47]. The mutant even remained essentially unaffected by the successive treatment with a hundredfold higher dose of cis- 22a, which led to an almost complete block of TRPV6 WT currents (Figure 2D).

Taken together, as shown for the G488R substitution, modifications within the TM4-TM5 linker which contributes to LBS-2 of TRPV6 may not only alter lipid binding and thus sensitivity to PIP_2_ depletion, but also sensitivity to pharmacological inhibition. Considering concomitant binding of cis-22a to LBS-2 and the pore of the TRPV6 WT channel, with the latter serving as the main binding site, the strong reduction in inhibition seen for the TRPV6 G488R mutation indicates that LBS-2 allosterically influences the pore, what is likely also relevant in the absence of the blocker, as supported by structural data on open and closed TRPV6 channels [45,46]. Although we are aware that the binding sites within the G488R variant and the TRPV6 WT channel are not necessarily the same, this prompted us to set out a screen for LBS-2 mutants showing a reduced sensitivity to cis-22a treatments and to test whether these mutants behave differently upon applying the pal-PIP_2_ peptide as well.

### 2.3. Inhibition of TRPV6 WT by cis-22a Shifts Constitutive Nuclear NFAT Localization to a Mainly Homogeneous Distribution

**Figure 3:**
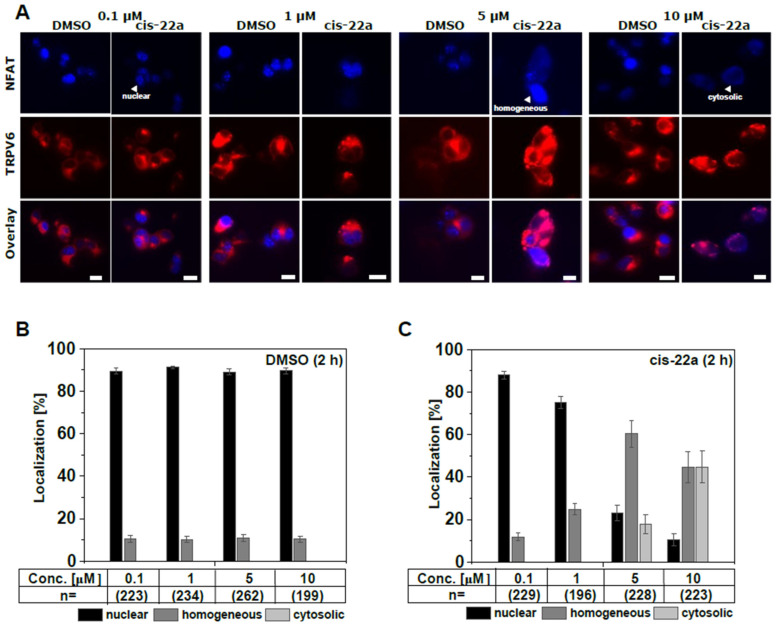
Cis-22a-based inhibition of TRPV6 WT reduces NFAT basal nuclear localization in a dose-dependent manner. (**A**) Exemplary fluorescence images on HEK293 cells co-transfected with N-terminally CFP-tagged NFAT (cyan) and TagRFP-labeled TRPV6 WT (red) after incubation for two hours with DMSO (solvent control) or cis-22a at increasing concentrations (0.1 μM to 10 μM). The scale bar indicates 10 μm. (**B**) Average (mean ± SEM) localization of NFAT in the nucleus (black), the cytosol (light grey), or an indifferent, homogeneous distribution after incubation with different concentrations of DMSO for 2 h serving as control for the measurements shown in (**C**), representing the mean (±SEM) localization of NFAT upon co-expression with TRPV6 WT following treatment with the indicated dose of cis-22a for two hours.

A straightforward way to analyze larger sets of mutants for functional alterations and/or sensitivity to pharmacological treatments is to monitor downstream responses such as transcription factor activation by either monitoring expression of a target gene or translocation of the respective transcription factor [64,65,66,67,68,69]. One downstream response to TRPV6-mediated Ca^2+^ influx is a translocation of transcription factors of the nuclear factor of activated T-cells (NFAT) family into the nucleus [70,71,72]. In contrast to other types of channels [64,65,73], co-transfection of HEK293 cells with constitutively active TRPV6 and NFAT targets the latter protein to the nucleus under basal conditions (Supplementary Figure S2A,B). Therefore, we wondered whether this localization pattern indicative of channel activity shifts upon incubation with cis-22a, and if so, whether NFAT localization might serve as a read-out for mutation-based changes in response to the inhibitor. To establish appropriate treatment conditions, HEK293 cells co-transfected with TagRFP-labeled TRPV6 WT and CFP-tagged NFAT were treated with different concentrations of cis-22a or with the equivalent concentrations of DMSO as solvent control for two hours. Thereby, three different localization patterns were apparent: nuclear, cytosolic, and homogeneous, with fluorescence emission occurring indifferently throughout the entire cell. At any concentration tested, the solvent control led to rather similar localization patterns as seen in the untreated state, connoting about 90% nuclear NFAT and about 10% of cells showing a homogeneous distribution but no complete cytosolic residence (Figure 3A–C, Supplementary Figure S2B). This distribution pattern was essentially preserved upon treatment with 0.1 μM cis-22a (Figure 3C). However, increasing the dose of the blocker to 1, 5, or 10 μM continuously decreased the proportion of cells showing nuclear NFAT localization but favored a homogeneous distribution. Cytosolic localization was only observed at the two highest concentrations tested, whereby the likelihood for homogeneous and cytosolic localization equalized at a dose of 10 μM cis-22a (Figure 3C).

Apart from dosage, incubation time appeared as a critical determinant for NFAT localization, whereby only a small proportion of cells maintained nuclear localization after incubation with 5 μM cis-22a for three or four hours, while cytosolic residence considerably increased to roughly 40%. Interestingly, distribution patterns were rather similar for incubation times of one and two hours, as well as for three and four hours, respectively, but showed marked differences between these two groups (Supplementary Figure S2C,D).

Given the strong effect of cis-22a treatments on subcellular NFAT targeting and having established channel inhibition by the pal-PIP_2_ peptide in electrophysiological measurements as well as in Ca^2+^-imaging experiments, we wondered whether also PIP_2_ sequestration was competent of shifting NFAT localization. However, even a rather high concentration of 10 μM pal-PIP_2_ peptide, inhibiting TRPV6 WT by about 56% in patch clamp measurements (Figure 1C,D, Supplementary Figure S1B), was unable to change localization patterns at different incubation times (Supplementary Figure S2E,F). We refrained from further increases in the concentration due to concomitant increases in DMSO levels. Considering that NFAT localization was also not affected by a dose of 0.1 μM cis-22a (Figure 3C), leading to a similar level of current inhibition as 10 μM pal-PIP_2_ peptide in patch clamp measurements (Figure 1C,D and Figure 2D), it seems that halving TRPV6 currents is not enough to impede NFAT activation. Apart from that, the peptide might have been subject to degradation upon prolonged incubation, e.g., by endogenous peptidases [74] and the levels of the lipid within the plasma membrane are impacted by ongoing PIP_2_ re-synthesis.

Taken together, TRPV6 WT fosters constitutive nuclear NFAT localization which is, after cis-22a treatment and depending on the concentration and incubation time, reverted to a predominantly homogenous distribution.

### 2.4. Site-Directed Mutagenesis in LBS-2 Alters Inhibition by cis-22a and Sensitivity to PIP_2_ Depletion

**Figure 4:**
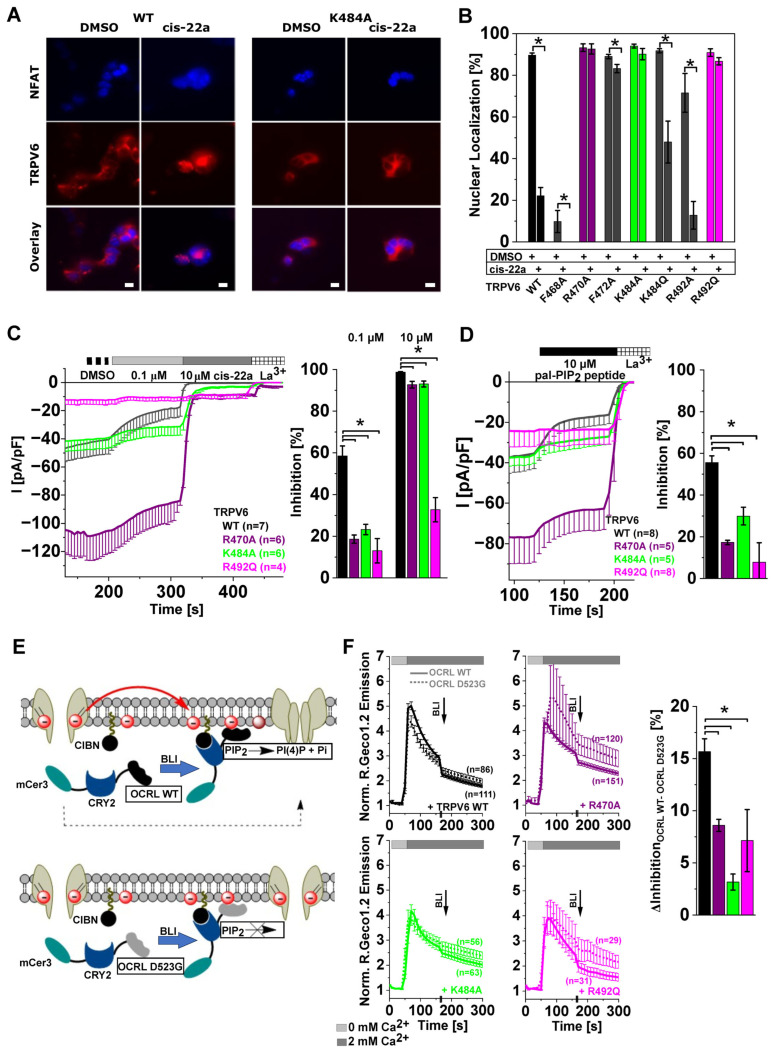
Point mutations within the extended TM4-TM5 linker/LBS-2 reduce channel sensitivity to PIP_2_ depletion and lower inhibition by cis-22a. (**A**) Representative fluorescence images on HEK293 cells co-transfected with CFP-NFAT (cyan) and TagRFP-labeled TRPV6 (red) WT (**left**) or TRPV6 K484A (**right**) recorded after incubation for two hours with DMSO or 5 μM cis-22a. The scale bar indicates 10 μm. (**B**) Average (mean ± SEM) of cells showing a nuclear localization of NFAT upon co-expression with TRPV6 WT or the indicated mutants after treatment with DMSO or 5 μM cis-22a for two hours. Asterisks (*) indicate statistically significant differences (*p* < 0.05) in nuclear localization between both treatments. (**C**) (**Left**): Mean (−SEM) whole-cell current density of TRPV6 WT/R470A/K484A or R492Q expressing HEK293 cells recorded in 10 mM Ca^2+^ solution successively supplemented with DMSO, 0.1 μM cis-22a, 10 μM cis-22a, and 1 mM La^3+^, as indicated. (**right**): Corresponding mean (±SEM) levels of inhibition reached by 0.1 μM and 10 μM cis-22a relative to the La^3+^ block. (**D**) Time traces (mean − SEM) of whole-cell current densities of HEK293 cells transfected with the same constructs as in (**C**) upon treatment with 10 μM pal-PIP_2_ peptide (**left**) and the corresponding values of inhibition (**right**). (**E**) Scheme on the components and principle of the optogenetic PIP_2_ depletion system. Blue light illumination (BLI) triggers interactions between CRY2 and prenylated, thus membrane-anchored, CIBN, which brings the CRY2-linked phosphatase domain of OCRL to the membrane. This localization enables PIP_2_ depletion by OCRL WT (**upper panel**) but not in the case of the catalytically inactive D523G mutant (**lower panel**). (**F**) (**left**): Time traces of normalized R.Geco1.2 intensities (mean ± SEM) derived from HEK293 cells co-expressing TRPV6 WT or the indicated mutant with the optogenetic PIP_2_ depletion system including either OCRL WT (solid line) or OCRL D523G (dashed line). CRY2-CIBN interactions were triggered by illumination with 475 nm for 30 s, as indicated by the arrow. The extents of reduction in signal intensity were determined for both constellation (OCRL WT or D523G) for each TRPV6 construct and the reduction derived upon co-expression of the inactive OCRL mutant was consequently subtracted from the value determined for OCRL WT to yield channel inhibition due to phosphatase activity ((**right**); mean ± SEM). For the shown patch clamp and Ca^2+^ imaging data, asterisks (*) indicate statistical significance between TRPV6 WT and mutants (*p* < 0.05).

Upon preparing a library of LBS-2 mutants to be investigated in NFAT measurements for eventual effects on the pore, we first focused on positions which have already been implicated in lipid binding, either via direct interactions or by contributing to suggested mechanisms relaying the signal of lipid binding to the pore [46,54,56,58]. For the actual screening efforts, changes in nuclear NFAT localization occurring after treatment with 5 μM cis-22a for 2 h were monitored. While a significant difference in nuclear NFAT localization was seen when incubating TRPV6 WT overexpressing HEK293 cells under the mentioned conditions with cis-22a (22% nuclear) compared to the DMSO control (90% nuclear), the analogous treatment failed to significantly reduce NFAT nuclear localization when co-expressed with the charge-neutralized LBS-2 mutants TRPV6 R470A, TRPV6 K484A, and TRPV6 R492Q (Figure 4A,B, Supplementary Figure S3A). Also, the TRPV6 G488R variant impeded cis-22a-based reductions in nuclear NFAT localization (Supplementary Figure S3B), consistent with the already presented patch clamp data on the weaker inhibition by cis-22a (Figure 2D). Of note, a reduced nuclear NFAT localization already in the absence of cis-22a was observed for the mutants TRPV6 F468A and TRPV6 R492A. Both mutants hindered in NFAT activation in the absence of cis-22a subserved a further significant reduction in nucleus-targeted NFAT after incubation with cis-22a.

TRPV6 LBS-2 mutants which showed complete unresponsiveness to cis-22a treatment in the NFAT screen were consequently characterized more closely in patch clamp measurements. Thereby, some mutants which were investigated in our previous publications [47,62] are shown again to simplify comparison with other treatments. Current inhibition of TRPV6 R470A [47], TRPV6 K484A [62], and TRPV6 R492Q was significantly reduced upon applying 0.1 μM cis-22a. Successive perfusion of 10 μM cis-22a, a dose which almost completely (~98%) impedes TRPV6 WT currents, also culminated in a significantly reduced inhibition of the mutants (Figure 4C), which is in line with the data on NFAT localization. In analogy to the TRPV6 G488R mutant showing both a reduced response to cis-22a as well as to the pal-PIP_2_ peptide (Figure 2B,D, Supplementary Figure S1E), the three charge-neutralized LBS-2 variants, i.e., TRPV6 R470A, TRPV6 K484A, and TRPV6 R492Q, also further displayed a significantly reduced sensitivity to peptide-based PIP_2_ sequestration (Figure 4D). Interestingly, in electrophysiological measurements, the relative extent of inhibition by the lower concentrations of cis-22a and by 10 μM pal-PIP_2_ peptide of the investigated LBS-2 mutants even followed the same profile.

As an alternative means of rapid PIP_2_ depletion which has, in the TRP field so far, just been applied to the study of TRPC channels [75,76], we took advantage of a light-activated phosphatase system developed by Idevall-Hagren and colleagues (2012) [1]. Apart from TRPC4 and TRPC5 homomeric channels, as well as TRPC1/4 or TRPC1/5 heterotetramers [75,76], the concerning system has already been implemented to modulate KCNQ2/3 channel activity [1] and, among others, the epithelial sodium (Na^+^) channel ENaC [77,78] or NaV1.4 [79]. The respective approach is based on the reversible dimerization between the plant-derived proteins cryptochrome 2 (CRY2) and the transcription factor CIB1 (cryptochrome-interacting basic helix–loop–helix 1) upon blue light illumination (BLI; ~440–490 nm) [1,79]. To be exploited for light-induced PIP_2_ depletion, the segment of CRY2 which senses light and consequently undergoes a conformational change, fostering its association with the N-terminal region of CIB1 (CIBN), has been linked to the inositol 5′-phosphatase domain of OCRL (oculocerebrorenal syndrome of Lowe). Instead, the CIBN component is fused to a prenylation motif, the CAAX box, at its C-terminus so that it becomes constitutively anchored to the plasma membrane. Thus, BLI-induced binding of CRY2 to CIBN concomitantly recruits the CRY2-linked phosphatase domain to the plasma membrane, where it converts PIP_2_ to phosphatidylinositol-4-phosphate (PI(4)P) by cleaving off the 5′-phosphate moiety (Figure 4E, upper panel) [1,78]. PI(4)P does not activate TRPV6 at physiologically relevant concentrations [19,59]. For control measurements, a D523G mutation was introduced into the 5′-phosphatase domain to render the OCRL component enzymatically inactive [1], while translocation after BLI remains intact (Figure 4E, lower panel). Interestingly, OCRL itself was already reported to endogenously regulate TRPV6 activity by both reducing PIP_2_ levels via its 5′-phosphatase domain as well as by modulating membrane trafficking in dependence of its Rab binding domain, the latter of which is, however, not covered by the fragment contained in the optogenetic PIP_2_ depletion system [1,80].

Upon testing different conditions for activation, exposure of accordingly transfected HEK293 cells to 475 nm for 30 s resulted in discernible dissociation of a fluorescent PIP_2_ sensor, an mCherry-labeled pleckstrin homology (PH) domain, from the plasma membrane (Supplementary Figure S4A). While this points to reductions in PIP_2_ levels and thus to an effective activation of the optogenetic system, translocation of the mCherry-labeled form of the CRY2-linked phosphatase to the plasma membrane was not that clearly visible (Supplementary Figure S4B), as already experienced by others [78]. To enable comparison of subcellular localization before and after BLI, translocation analysis harnessed red-shifted fluorescent labels to avoid pre-activation during cell search, considering the absorption spectrum of CRY2 [81,82]. Consistently, the genetically encoded indicator R.Geco1.2 was used for Ca^2+^ imaging to investigate the effects of optogenetic PIP_2_ depletion on TRPV6 activity. For TRPV6 WT, exposure to 475 nm for 30 s resulted in a marked decrease in the R.Geco1.2 emission intensity (Figure 4F). To differentiate between the effects of BLI-triggered PIP_2_ depletion and reductions in signal intensity due to bleaching of the indicator or caused by an inherent time-dependent decrease in channel activity, measurements were conducted in parallel with the catalytically inactive OCRL D523G variant [1], leading only to a modest reduction after BLI upon co-expression with TRPV6 WT (Figure 4F, upper left panel). In line with reduced inhibition by the pal-PIP_2_ peptide, the difference in the decrease upon co-expressing TRPV6 LBS-2 mutants with either OCRL WT or OCRL D523G was significantly lower compared to TRPV6 WT (Figure 4F, right). The TRPV6 G488R mutant was harnessed as control for the light-sensitive phosphatase system as well, given that a higher PIP_2_ affinity of this variant is backed up by multiple lines of experimental evidence [59]. Indeed, the mutant was largely resistant to optogenetic PIP_2_ depletion, either as about the same reduction in R.Geco1.2 emission intensity occurred upon co-expressing OCRL WT or the inactive D523G mutant with TRPV6 G488R (Supplementary Figure S4C).

All in all, the reduced sensitivity of TRPV6 upon mutating the residues R470, K484, or R492 (or also G488) within LBS-2 to cis-22a treatments, to the pal-PIP_2_ peptide, and to optogenetic PIP_2_ depletion corroborates the importance of this site for channel regulation. Moreover, it further supports the notion that both regions within the channel, i.e., LBS-2 and the pore, are not acting independently of one another.

### 2.5. TRPV6 Current Inhibition by cis-22a and PIP_2_ Depletion Is Sensitive to Site-Directed Mutagenesis within the Ion Permeation Pathway

**Figure 5:**
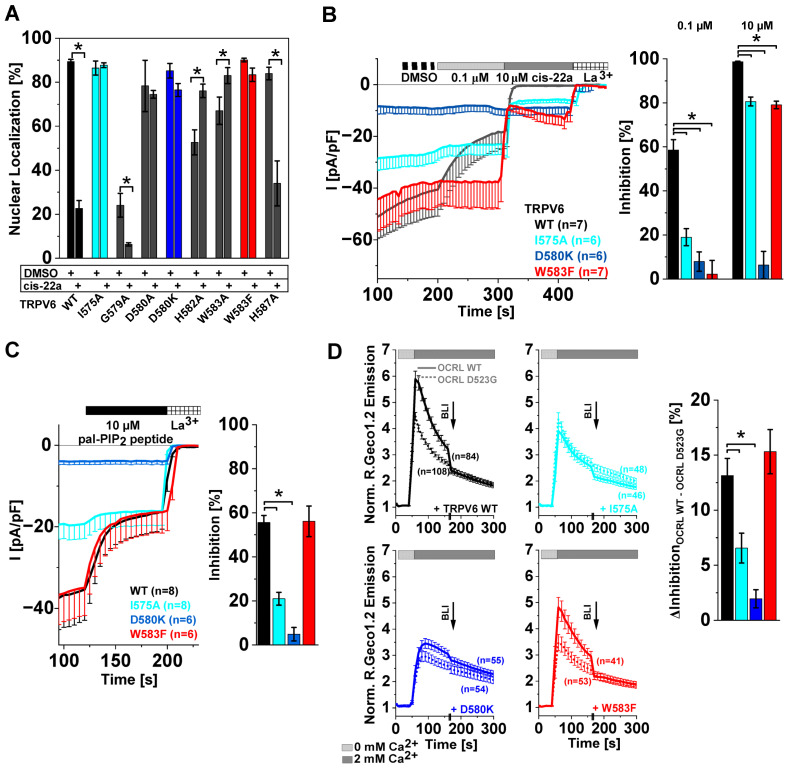
A reduction in the inhibitory potential of cis-22a on TRPV6 pore mutants does not necessarily go along with a lower sensitivity to PIP_2_ depletion. (**A**) NFAT screening data on nuclear localization of CFP-tagged NFAT in HEK293 cells expressing TRPV6 pore mutants and the wildtype control after incubation with 5 μM cis-22a or analogously diluted DMSO for two hours. The asterisk (*) indicates a statistically significant difference in nuclear NFAT localization between both treatments for the respective TRPV6 construct. (**B**) (**left**): Average (mean − SEM) whole-cell current density recorded in HEK293 cells expressing TRPV6 WT or the pore mutants TRPV6 I575A, TRPV6 D580K, or TRPV6 W583F in 10 mM Ca^2+^ solution upon consecutive addition of DMSO, 0.1 μM cis-22a, 10 μM cis-22a, and 1 mM La^3+^. (**right**): Mean (±SEM) levels of inhibition reached by 0.1 μM and 10 μM cis-22a, corresponding to the time traces. (**C**) Whole-cell patch clamp-based analysis of the sensitivity of TRPV6 I575A, TRPV6 D580K, TRPV6 W583F, and the wildtype control to PIP_2_ sequestration by 10 μM pal-PIP_2_ peptide ((**left**): time traces, mean − SEM; (**right**): mean percent of inhibition by 10 μM pal-PIP_2_ peptide ± SEM). (**D**) Ca^2+^ imaging data (normalized mean R.Geco1.2 emission ± SEM) on optogenetic PIP_2_ depletion in HEK293 cells upon co-expressing TRPV6 WT or the indicated mutant with prenylated CIBN, CRY2-OCRL WT (solid lines), or the D523G mutant (dashed lines), respectively. The difference in inhibition in the presence of OCRL WT and OCRL D523G derived from the time traces on the left was used to evaluate TRPV6 inhibition by phosphatase activation (right, mean ± SEM). For the shown patch clamp data and R.Geco1.2 measurements, asterisks (*) indicate statistical significance between TRPV6 WT and mutants (*p* < 0.05).

Given an apparent allosteric communication of LBS-2 with the pore, we wondered whether such an interplay is also present in the reverse direction and if mutations within the pore might alter responses to cis-22a and lipid depletion in a similar manner as described before for LBS-2. To investigate this, we harnessed the same strategy as for LBS-2, namely to first screen for pore mutants preserving nuclear NFAT localization after incubation with 5 μM cis-22a for 2 h and to characterize these more closely in follow up experiments. Upon mutating residues in TM6, the proportion of cells showing NFAT predominantly in the nucleus was not significantly changed following cis-22a treatment when co-expressing the TRPV6 mutants I575A, D580A, D580K, or W583F, respectively. Considering the TRPV6 G579A substitution, a homogeneous rather than nuclear localization of NFAT was preferred already in the absence of cis-22a, while treatment with the blocker led to a further significant reduction in the proportion of cells showing nucleus-resident NFAT (Figure 5A, Supplementary Figure S5A). For TRPV6 H582A and TRPV6 W583A, nuclear NFAT localization was higher for cis-22a-treated cells compared to the DMSO control (Figure 5A, Supplementary Figure S5A). We decided not to pursue this aspect any further, as cells appropriate for analysis were hardly present when expressing any of these mutants. Indeed, strongly reduced cell viability was already reported earlier for homologous TRPV5 W583A channels [83,84].

Moreover, substitutions were introduced farther towards the C-terminus at positions proposed by Cai et al. (2020) [58] to either directly bind to PIP_2_ (TRPV6 R589, TRPV6 R632) or to be involved in the mechanism of PIP_2_-based channel activation (TRPV6 W593). For TRPV6 R632A/Q, the reduction in nuclear NFAT due to cis-22a incubation was similar to that determined for TRPV6 WT, while TRPV6 W593A led to a lower yet significant decrease in the proportion of cells showing NFAT in the nucleus after cis-22a incubation (Supplementary Figure S5B). Nuclear targeting of the transcription factor was almost absent for TRPV6 R589A for both the solvent control and blocker-treated cells (Supplementary Figure S5C).

The featured remarkable resistance to cis-22a-based reductions in nuclear NFAT of TRPV6 I575A, TRPV6 D580K, and TRPV6 W583F goes along with a significantly reduced response to the blocker in patch clamp measurements (Figure 5B) [47]. Reportedly, engineering the channel with the W583F mutation leads to an almost hundred-fold increase in the IC_50_ value from 82 nM for TRPV6 WT to ~6.19 μM in the case of the mutant [47]. Apart from inhibition by cis-22a, I575A and D580K substitutions within the pore interfered with the inhibitory effect of the pal-PIP_2_ peptide (Figure 5C), in analogy to LBS-2 mutants. Surprisingly, however, the sensitivity of TRPV6 W583F to peptide-based PIP_2_ sequestration was not changed compared to the wildtype control. Investigations of the same mutants with the light-sensitive phosphatase system corroborated the reduced sensitivity of the TRPV6 I575A and TRPV6 D580K mutant to PIP_2_ depletion and substantiated the wildtype-like response of the TRPV6 W583F variant (Figure 5D).

In summary, our screen on selected LBS-2 and pore mutants showed that single substitutions within one of these two functionally important regions may concomitantly alter responses to PIP_2_ depletion and cis-22a. For mutations within the pore, a reduced sensitivity to cis-22a is consistent with the pore serving as the main binding site of the inhibitor [47], whereas a lower inhibition by PIP_2_ sequestration or depletion seems to indicate that LBS-2 and the pore are allosterically coupled in a bidirectional rather than monodirectional manner. Yet, one of the analyzed mutants, TRPV6 W583F, was largely resistant to inhibition by cis-22a but remained sensitive to optogenetic depletion or peptide-based sequestration of PIP_2_ like the wildtype control, indicating that mechanistic differences exist.

### 2.6. Differences in TRPV6 Channel Inhibition by cis-22a and PIP_2_ Depletion

**Figure 6:**
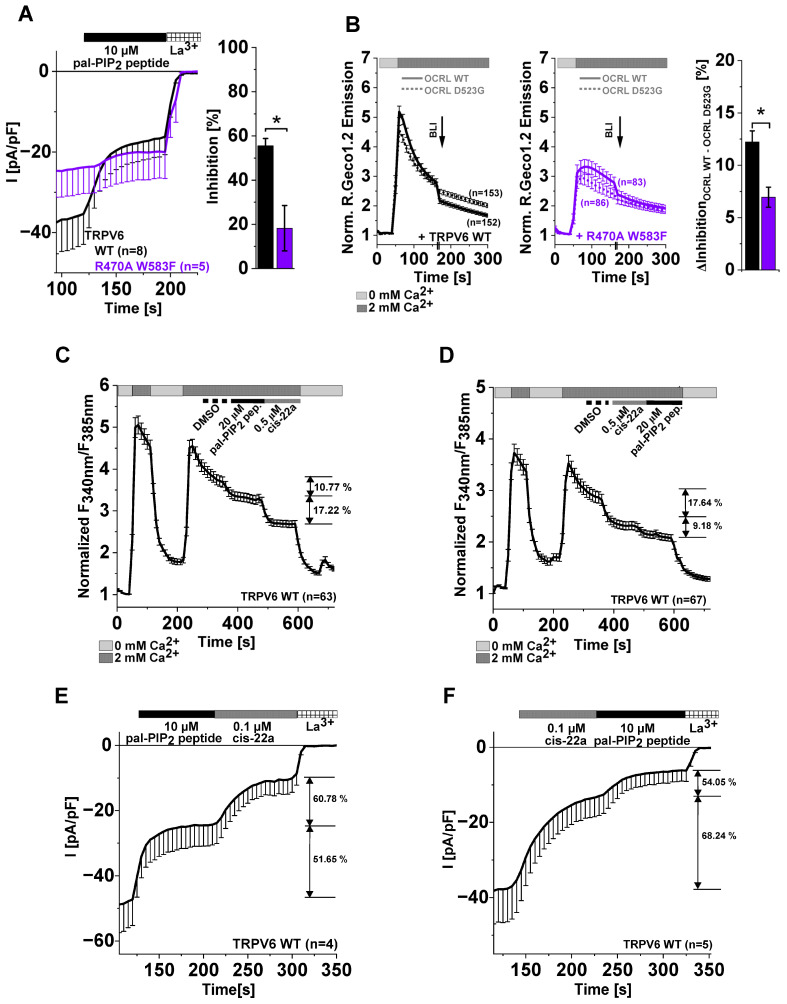
Pretreatment of TRPV6 with pal-PIP_2_ peptide or cis-22a does not alter inhibition by the consecutively added other compound. (**A**) Whole-cell current densities (mean − SEM) of HEK293 cells transfected with TRPV6 WT or the LBS-2/pore double mutant TRPV6 R470A W583F upon treatment with 10 μM pal-PIP_2_ peptide (**left**) and the extents of inhibition therefrom derived relative to the La^3+^ block (**right**). (**B**) Normalized R.Geco1.2 emission intensities (mean ± SEM) derived from measurements on HEK293 cells co-expressing TRPV6 WT (**left**) or TRPV6 R470A W583F (**middle**) with the optogenetic PIP_2_ depletion system, including either OCRL WT (solid line) or the inactive control OCRL D523G (dashed line). The differences in signal reduction after blue light illumination (BLI, 475 nm 30 s) between the measurements made in the context of OCRL WT and OCRL D523G for TRPV6 WT and the mutant are shown on the (**right**). For the bar charts in (**A**,**B**), the asterisk (*) indicates statistical significance (*p* < 0.05) between the TRPV6 WT control and the mutant. (**C**) Normalized Fura-2 measurements (mean ± SEM) on HEK293 cells overexpressing TRPV6 WT upon consecutive perfusion with 20 μM pal-PIP_2_ peptide and 0.5 μM cis-22a following the DMSO solvent control, as indicated by the bars. The mean percentage of inhibition by the pal-PIP_2_ peptide and cis-22a is highlighted by arrows. (**D**) Analogous measurement to (**C**) with the reverse order of cis-22a and pal-PIP_2_ peptide application. (**E**) Whole-cell current densities (mean − SEM) of HEK293 cells transfected with TRPV6 WT upon treatment with 10 μM pal-PIP_2_ peptide followed by 0.1 μM cis-22a and 1 mM La^3+^; the corresponding measurements, including the reverse order of application (0.1 μM cis-22a, 10 μM pal-PIP_2_ peptide), are shown in (**F**). In both graphs, the mean percentage of inhibition by either compound is indicated by the arrows.

In our previous work, a very weak inhibition by cis-22a was observed when combining the W583F pore mutation with the R470A substitution in LBS-2 (TRPV6 R470A W583F), which led to the first speculations that the LBS-2 mutation might impose allosteric effects on the pore binding site of the blocker [47]. As both single mutations interfered with inhibition by cis-22a but only the LBS-2 mutation reduced sensitivity to both approaches, reducing the level of free PIP_2_ within the membrane (Figure 4C,D,F and Figure 5B–D), we were interested in the response of the TRPV6 R470A W583F double mutant when reducing PIP_2_ availability. NFAT localization was examined as well, whereby incubation of HEK293 cells expressing TRPV6 R470A W583F with 5 μM cis-22a for 2 h was unable to change localization patterns (Supplementary Figure S6A), consistent with the just modest inhibition observed in electrophysiological measurements (Supplementary Figure S6B) [47]. Contrasting the pore-only mutant (TRPV6 W583F), channels carrying W583F and R470A substitutions at the same time regained significant resistance to inhibition by the pal-PIP_2_ peptide and OCRL-based PIP_2_ depletion (Figure 6A,B) and were thus in this regard more reminiscent of the R470A single mutant.

To shed further light on channel modulation by both cis-22a treatment and PIP_2_ depletion, we sought to investigate whether consecutive application of the pal-PIP_2_ peptide and cis-22a or vice versa leads to alterations in the respective extent of inhibition. Assuming that the same mechanism would underlie both inhibitory processes, one could expect that, altogether, no additional inhibition would occur at the second step or that inhibitory levels would differ between both application orders. Therefore, comparatively low concentrations of cis-22a were implemented to facilitate analysis of inhibition of the remaining current. Contrasting the stated hypothesis, in Fura-2 measurements, the signal decreased by ~11% when treating cells with 20 μM pal-PIP_2_ peptide before applying cis-22a (Figure 6C) and a statistically not significantly different reduction of ~9% was observed when the same dose of the peptide was added after 0.5 μM cis-22a (Figure 6D). The mean extent of inhibition by 0.5 μM cis-22a also remained the same, irrespective of whether the pal-PIP_2_ peptide was already added or not, amounting 17.22% and 17.64%, respectively (Figure 6C,D). Considering patch clamp recordings, treatment with 10 μM pal-PIP_2_ peptide before 0.1 μM cis-22a inhibited TRPV6 currents by ~52%, and the residual current was further reduced by ~61% after blocker addition (Figure 6E). Also, in patch clamp experiments, a change in the order of application was unable to alter inhibitory levels of cis-22a and peptide-based PIP_2_ depletion in a statistically significantly manner, reaching about 68% by cis-22a and 54% by the pal-PIP_2_ peptide (Figure 6F). To ensure that the already added compound was not washed out by consecutively administering the other, we conducted control experiments by perfusing analogously diluted DMSO in the second step, whereby no re-activation occurred, neither for cis-22a nor for the pal-PIP_2_ peptide (Supplementary Figure S6C,D).

Taken together, our data on single LBS-2 and pore mutants as well as on channels concurrently carrying R470A and W583F substitutions point to a bidirectional allosteric communication between both sites of the channel. Notably, this bidirectionality is not fulfilled at the cytosolic end of the pore, as the TRPV6 W583F single mutant maintained wildtype-like sensitivity to PIP_2_ depletion.

## 3. Discussion

**Figure 7:**
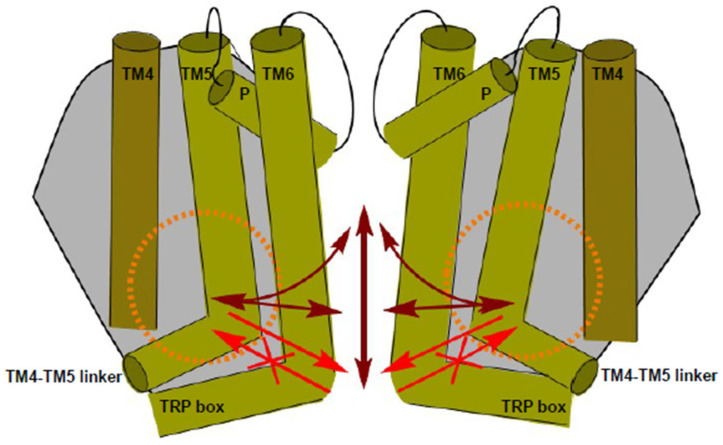
Position-dependent bidirectional allosteric coupling between LBS-2 and the pore. LBS-2 (orange, dashed circle) influences upper and lower regions of the pore. Allosteric communication exists also along the ion permeation pathway and while upper loci within the pore affect LBS-2, the cytosolic exit site TRPV6 W583 is devoid of retrograde coupling to LBS-2, as indicated by the arrows.

The membrane lipid phosphatidylinositol-(4,5)-bisphosphate (PIP_2_) is an important regulator of TRPV6 activity. Here, we show that the PIP_2_ binding site of TRPV6 is allosterically and in a bidirectional manner coupled to the pore domain and the permeation path of the channel. Our conclusions are based on the determination of the sensitivity of LBS-2 and pore mutants (Supplementary Figure S7A,B) to PIP_2_ depletion and cis-22a. Importantly, bidirectionality is observed for most but not all residues tested, since it appears absent at the intracellular exit of the pore (Figure 7).

This is substantiated by mutating the TRPV6 W583 residue to a phenylalanine, which left inhibition by PIP_2_ depletion unaffected but remarkably decreased inhibition by cis-22a. By contrast, sensitivity to PIP_2_ depletion was clearly reduced upon combination with an LBS-2 mutation (TRPV6 R470A W583F). Moreover, the considerably higher resistance of the LBS-2/pore double mutant TRPV6 R470A W583F to cis-22a compared to the one of the respective single mutants, especially at the high blocker concentration, corroborates the concept of allosteric coupling between both regions of the channel [47].

Previous structural data showed that cis-22a binds to LBS-2 and to the pore of the TRPV6 channel, with functional analysis depicting the pore to serve as the main binding site for cis-22a. This is explained by the overall higher resistance of the pore rather than LBS-2 mutants to the inhibitor [47]. Insertion of cis-22a into the pore is reminiscent of calmodulin (CaM) binding [47,85]. In both cases, characteristics of the open state, such as an α-to-π helical transition in TM6, are preserved. Nonetheless, the lower parts of TM6 are positioned closer to the pore axis compared to the open state, likely based on attractive forces imposed by the bound ligand [47]. Also, for CaM as well as for cis-22a, the four W583 residues of TRPV6 form a tight cubic cage around the respective ligand (Supplementary Figure S7C), with the specific arrangement supporting strong interactions [47,85]. Considering that the ion permeation pathway is essentially plugged by the pore-bound cis-22a molecule, rather strong reductions in the extent of inhibition observed for LBS-2 mutants indicate that LBS-2 impacts the pore in an allosteric manner. This is further corroborated by the strong reduction in inhibition observed when inserting mutations above the critical W583 binding site, supporting the concept that allosteric coupling also exists along the pore. Altogether, mutations within both binding sites may not only alter binding of the inhibitor thereto but also interfere in allosteric communication between the different channel domains.

With respect to lipid regulation, the existence of allosteric coupling from the site of lipid biding to the pore has also been inferred from structural studies on TRPV5 and TRPV6 [46,54], yet functional data in support of this concept have to date been lacking. Considering the structure of rabbit TRPV5 channels coordinating short chain, soluble di-octanoyl PIP_2_ [54], binding of this PIP_2_ mimetic reportedly induces movement of the cytosolic end of TM6 in a pore-averted direction. This leads to a repositioning of the bulky side chain of the TRPV5 W583 residue out of the pore and shifting the TM4-TM5 linker outwards [54]. This rabbit TRPV5 structure was recently harnessed to generate a structural model of PIP_2_-bound TRPV6, again indicating that binding of the lipid is allosterically coupled to the pore, specifically affecting the selectivity filter and the hydrophobic gate [56], yet lacking experimental evidence. Upon preparing this manuscript, another rabbit TRPV5 channel structure was presented, depicting similar conformational changes upon association of the activator long-chain acyl-Coenzyme-A with the same binding site as the PIP_2_ analog [86].

While a definite binding site for PIP_2_ has, in structural studies [46,47,48,49,55], thus far not been localized within the TRPV6 channel complex, our data demonstrate the involvement of LBS-2 residues in the regulation of the channel by PIP_2_. This finding is in line with the larger size of the LBS-2 density in the open compared to the closed state of the channel [46]. The latter might indicate direct binding thereto, which was further consistent with its chemical features, involving the presence of several positively charged residues [46]. Of note, the TRPV6 R470E channel features a slower Ca^2+^ influx and structurally depicts a smaller non-protein density engaging LBS-2 as well as loss of open-state conformational characteristics of TM6, apparent as a reversion of the α-to-π helical transition, re-rotation of TM6, and closer positioning towards the pore axis [46]. The R470E mutation within the human TRPV6 protein thereby leads to similar structural features as observed in channels formed by highly conserved rat TRPV6 proteins, surprisingly depicting a closed conformation in the wildtype state [21,45].

With regard to the fact that the lipid density resolved in cryo-EM data from the human channel is hardly reconcilable with PIP_2_, it is important to consider that all TRPV6 cryo-EM structures were derived in the presence of rather high concentrations of cholesterol hemi succinate (CHS). This might have led to the replacement of the naturally bound lipid [46,47,48,49,50,55]. Moreover, such an exchange in channel-resident lipids forced by the experimental conditions might have led to some adaptions in the binding site itself.

In view of the role for channel activation, it is somewhat puzzling that both the removal (TRPV6 R470A, TRPV6 K484A, TRPV6 R492Q) and insertion (TRPV6 G488R) of positive charges within LBS-2 apparently reduced sensitivity to PIP_2_ depletion and that the TRPV6 G488R substitution, which reportedly increases affinity to the activating lipid PIP_2_ [59], leads to experimental detection of smaller currents. Interestingly, the latter effect has already been observed by Velisetty and colleagues (2016) and is likely related to the lacking inactivation of the TRPV6 G488R mutant [59,62], leading to Ca^2+^ overload and cell death if expression levels are too high. For some of the analyzed LBS-2 residues, previous experimental data (K484) [58] and the aforementioned structural model of PIP_2_-bound TRPV6 (R492) indicate their involvement in PIP_2_ binding. The model of Wang et al. (2023) [56] suggests that other residues also interact with either the head group or fatty acid chains of PIP_2_, which where thus included in our preliminary NFAT screen, whereon detailed elucidations are available in the supplementary information.

Yet, it is important to note that the analyzed LBS-2 residues are not necessarily involved in PIP_2_ binding itself but might rather contribute to the coupling between PIP_2_ binding and the pore. Several mechanisms have been proposed in previous studies to explain TRPV6 regulation by an activating lipid, and specifically by PIP_2_, which are barely consistent with one another [46,56,58]. Therefore, the residues W321, R470, I575, and W593 were proposed to be engaged in autoinhibitory interactions of the N-terminus or of the TM4-TM5 linker with residues of the TRP helix, respectively (W321-I597, R470-W593) [58]. These interactions were suggested to be released when PIP_2_ binds to the channel. For lipid binding itself, the TRPV6 residues K484, R589, and R632 were reported as indispensable in the same study [58]. Later, Cai et al. (2021) proposed R492 to interact with D580, imposing autoinhibitory effects on the channel by stabilizing a narrow pore conformation. Hindrance due to R492Q substitution was reported to render D580 and the nearby gate region more flexible so that the pore becomes wider [87]. While the role of PIP_2_ was neglected in this study, it is tempting to combine these findings with R492 as putative PIP_2_ binding partner, as observed in the modeled structure [56]. Consistently, one might hypothesize that this autoinhibition was released upon PIP_2_ binding. In the present experiments, the R492Q mutant, which is listed in the catalog of somatic mutations in cancer (COSMIC) database and predicted to be pathogenic [87], showed a trend towards a reduced steady-state current density before starting treatments (Figure 4C,D). This observation suggests altered PIP_2_ binding and/or hindrance in relaying lipid regulation to the pore domain. In this regard, it is also interesting to note that lipid metabolism and membrane composition in cancer cells is in many cases altered in comparison with the corresponding non-cancerous tissues [88,89,90].

While it seems inevitably required that binding of PIP_2_ induces changes in the pore’s geometry to alter ion conduction, our present results show that the pore affects lipidic regulation as well, given the reduced response to peptide-based PIP_2_ sequestration and optogenetic conversion of PIP_2_ to PI(4)P of the pore mutants TRPV6 I575A and TRPV6 D580K, respectively. For pore sites, the specific positions might alternatively ultimately transduce the signal of lipid loss to changes in ion currents, for instance, by constricting the permeation pathway. Indeed, I575 was found to form the narrowest part of the pore in the open [46] as well as inactivated state [84]. However, the position within the pore is decisive for whether or not a pore mutation is able to interfere with inhibition by lipid depletion, considering that sensitivity to PIP_2_ depletion was maintained by the TRPV6 W583F mutation at the cytosolic exit. Although we cannot exclude that mutations directly led to changes in channel conformation, as, for instance, reported for TRPV5 W583A [83], this might also explain why inhibition of TRPV6 WT by cis-22a and the pal-PIP_2_ peptide remained unchanged, irrespective of whether the other substance was previously added or not (Figure 6C–F). Considering the structural insights on open, closed, and cis-22a-bound TRPV6 channels, PIP_2_ depletion eventually led to channel inhibition due to a narrowing within the region of the hydrophobic gate, while cis-22a-bound channels are ultimately blocked at W583.

In this study, we were able to prove the applicability of infrequently used but highly advantageous approaches to interfere with channel–PIP_2_ interactions: peptide-based sequestration and optogenetic PIP_2_ depletion. Our electrophysiological dose–response analysis thereby yielded a maximum inhibition of TRPV6 currents by the pal-PIP_2_ peptide application of about 60%, while almost complete channel inhibition appears upon treatment with high doses of cis-22a. Different factors might contribute to the former plateau in inhibition. On the one hand, considering that Ca^2+^ influx via TRPV6 is likely to lead to enzymatic PIP_2_ depletion [19,20] and that all administrations occurred when TRPV6 currents had reached a constant level, TRPV6 might already have lost some of its bound PIP_2_. Also, it was intuitive that the affinities of the pal-PIP_2_ peptide as well as of TRPV6 for PIP_2_ are relevant in this regard. Undoubtedly, the equilibration between peptide-bound PIP_2_, channel-bound PIP_2_, and free lipid, together with the dynamic resynthesis of PIP_2_, eventually set constraints on the extent of lipid depletion achievable. This maximum in inhibition may further contribute to the observation that NFAT localization remained unchanged upon pal-PIP_2_ peptide application.

Despite limits in the maximum level of inhibition reached upon acute application and inefficiency upon prolonged incubation, our present study provides evidence that TRP channel regulation by PIP_2_ can be investigated using the pal-PIP_2_ peptide as a PIP_2_ scavenger, circumventing side effects induced by products of enzymatic reactions. Also, possible secondary targets of pharmacological interference in PIP_2_ synthesis and turnover are mitigated, in addition to peptide-based inhibition arriving faster. Therefore, optimization of such membrane-anchored peptides, for instance, by sequence modifications to increase affinity for PIP_2_ or by inserting light-responsive groups to switch between PIP_2_ binding and inactive states, is highly interesting for future investigations.

## 4. Materials and Methods

### 4.1. DNA-Constructs and Reagents

N-terminally TagRFP-labeled human TRPV6 (725 aa) was kindly provided by R. Bhardwaj and M. Hediger (University of Bern, Bern, Switzerland). This variant is translated from the first ATG codon and thus deviates from the non-ATG-initiated protein which is expressed in vivo (accession number NM_018646.6) by lacking 40 amino acids of the very N-terminus. For N-terminal labeling with EYFP, TRPV6 was cloned into pEYFP-C1 (Clontech) via EcoRI and SacII restriction sites. Mutations (F468A, R470A, F472A, K484A/Q, G488R, R492A/Q, I575A, G579A, D580A/K, H582A, W583A/F, H587A, R589A, W593A, R632A/Q, R470A, W583F) were introduced using the QuickChange XL site-directed mutagenesis kit (Stratagene, San Diego, CA, USA) and Pfu DNA Polymerase (Promega, Madison, WI, USA). pTagRFP-hTRPV6 I575A was kindly provided by the lab of M. Hediger (University of Bern, Bern, Switzerland). Forward and reverse primers for site-directed mutagenesis were synthesized by Eurofins genomics GmbH (Wien, Vienna, Austria). Intactness of the constructs in terms of sequence was confirmed by sequencing (Microsynth Austria GmbH, Wien, Vienna, Austria; Eurofins genomics GmbH, Wien, Vienna, Austria). In the present experiments, Tag-RFP-labeled constructs were implemented for NFAT screens, patch clamp measurements, and Fura-2-based Ca^2+^ imaging, while for R.Geco1.2 measurements, TRPV6 WT and mutants were used which carried an EYFP label at the N-terminus (pEYFP-C1 vector) to avoid spectral overlap. R.Geco1.2 was purchased from Addgene (No. 45494), and mCherry-CRY2-5ptase OCRL was established in the lab of P. De Camilli and was derived from Addgene (No. 66836), as was CIBN-CAAX (No. 79574). Plasmids encoding for mCerulean3-CRY2-5′ ptase OCRL, YFP-CIBN-CAAX, and the mCherry-tagged PH domain were provided by K. Groschner (Medical University of Graz, Graz, Austria). Catalytically inactive OCRL D523G mutants were generated in analogy to TRPV6 mutants. CFP-NFAT (NFATc1, equivalent to NFAT2) was kindly provided by R. Kehlenbach (Scripps Research Institute, La Jolla, CA, USA). The TRPV6 inhibitor, cis-22a, was synthesized and provided by the lab of Jean-Louis Reymond (University of Bern, Bern, Switzerland).

### 4.2. Peptide Synthesis, Purification, and Characterization

Peptides were prepared on TGS Wang resin (250 mg, capacity = 0.27 mmol/g) in an automated peptide synthesizer (Syro-I, Biotage, Uppsala, Sweden) using Fmoc/tBu strategy with DIC/HOBt coupling reagents. Palmitic acid was coupled on the N-terminus of the peptides using DIC/HOBt coupling reagents. After the synthesis was complete, peptides were cleaved from the resin with TFA in the presence of scavengers (H_2_O and TIS, 3–3 *v*/*v*%). Crude products were precipitated in cold diethyl ether, centrifuged (4000 rpm, 5 min), and lyophilized from H_2_O/AcN. Palmitoylated peptides were then purified by RP-HPLC on a Phenomenex Jupiter Proteo C12 column (10 μm, 90 Å, 10 mm × 250 mm) with linear gradient elution using 0.1% TFA in H_2_O (eluent A) and 0.1% TFA in AcN:H_2_O = 80:20 (*v*/*v*) (eluent B) on an UltiMate 3000 Semiprep HPLC (Thermo Fisher Scientific, Waltham, MA, USA). Purified peptides were analyzed by RP-HPLC using an LC-40 HPLC System (Shimadzu, Kyoto, Japan) on an analytical Phenomenex Jupiter Proteo C12 column (10 μm, 90 Å, 4.6 mm × 150 mm). The flow rate was 1 mL/min, and the gradient was 5–100 B% in 20 min (UV detection at λ = 220 nm). High-resolution mass spectra were acquired by direct injection to a Thermo Scientific QExactive Focus Hybrid Quadrupole-Orbitrap Mass Spectrometer (Waltham, MA, USA). Data were analyzed by Xcalibur program (Thermo Fisher Scientific, Waltham, MA, USA).

### 4.3. Cell Culture and Transfection

Cultivation of human embryonic kidney (HEK) 293 cells occurred in DMEM supplemented with 10% fetal calf serum, penicillin (100 U/mL), and streptomycin (100 µg/mL) at 37 °C under humidified atmosphere containing 5% CO_2_. Transient transfection was performed 20–24 h prior to experiments using the TransFectin lipid reagent (Bio-Rad, Hercules, CA, USA), in accordance with the instructions of the supplier. For Ca^2+^ imaging using R.Geco1.2, 2 μg of the indicator-encoding plasmid were additionally transfected, while 1 μg plasmid DNA was routinely used per construct in all other cases. For patch clamp experiments, cells were reseeded on poly-L-lysine-coated glass slides at least 4 h prior to experiments. To exclude mycoplasma contaminations, culture supernatants were regularly tested by means of the VenorGem Advanced Mycoplasma Detection kit (VenorGEM, Berlin, Germany).

### 4.4. Electrophysiology

Electrophysiological experiments were performed at 20 °C to 24 °C in whole-cell recording configurations of the patch clamp technique. A Ag/AgCl electrode was used as a reference electrode. The implemented voltage ramp protocol included a holding potential of +50 mV, wherefrom voltage was ramped from −90 mV to +90 mV within 200 ms with an interval of 5 s between individual ramps. For the time courses, the inward current density at −74 mV was considered. The internal pipette solution contained 145 mM cesium methane sulfonate, 8 mM NaCl, 3.5 mM MgCl_2_, 10 mM HEPES, and 20 mM EGTA (pH 7.2). For electrophysiological measurements, the basic extracellular solution was composed of 145 mM NaCl, 5 mM CsCl, 1 mM MgCl_2_, 10 mM HEPES, 10 mM glucose, and 10 mM CaCl_2_ (pH 7.4). Leak correction of currents was performed by subtracting the residual current after 1 mM La^3+^ application at the end of the experiment. The liquid junction potential was determined as 12 mV; the voltages were not adjusted. Clampfit 11.1.0.23 and OriginPro 2020b software were used for data analysis. All experiments were conducted on a minimum of two different days.

### 4.5. Fura-2 Measurements

HEK293 cells were grown on poly-L-lysine-coated glass slides for 24 h before transfection. On the day after transfection, coverslips were cleared from medium remnants and detached cells upon washing with nominally Ca^2+^-free extracellular solution (0 mM Ca^2+^ ECS; 140 mM NaCl, 5 mM KCl, 1 mM MgCl_2_, 10 mM HEPES, 10 mM glucose, pH adjusted to 7.4 with NaOH). Thereafter, cells were loaded with 1 μM Fura-2-AM (SIGMA Life Science, Taufkirchen, Germany, 47989-1MG-F) in standard Ca^2+^-free solution (0 mM Ca^2+^ ECS) at room temperature in darkness. Fura-2 stock solutions were prepared at a concentration of 1 mM in dimethyl sulfoxide (DMSO; Merck, Darmstadt, Germany) without further additions. After 25 min, the dye-loading solution was removed, the cell layer was again carefully rinsed with 0 mM Ca^2+^ ECS, and incubation was continued for another 25 min to allow for complete de-esterification. Glass slides were consequently mounted on an Axiovert 135 inverted microscope (Zeiss, Oberkochen, Germany), subserving a sCMOS-Panda digitale Scientific Grade camera 4.2 MPixel and a LedHUB LED Light-Engine (LedHUB^®^; Omicron-Laserage Laserprodukte GmbH, Rodgau, Germany), manipulation of which occurred via Omicron Control Center v3.9.28. A CHROMA 69,008× ET ECFP/EYFP/mCherry 25 mm Dia Mounted filter was installed to search TagRFP-hTRPV6 expressing cells, while a FURA dichroic mirror (OMEGA OPTI-CAL XF114) was used for calcium imaging. Experiments were carried out using the VisiView 4.5.0 software (Visitron Systems GmbH, Puchheim, Germany). Successfully transfected cells were marked as regions of interest for data recording. Throughout measurements, Fura-2 was alternatingly excited at 340 nm and 385 nm with an exposure time of 100 ms each and an interval between successive iterations of 10 s. For both excitation wavelengths, emission was monitored at 505 nm and the ratio of the emission intensities detected upon excitation at 340 nm and 385 nm was determined by the VisiView software after background subtraction. In the course of the measurement, cells were initially perfused with Ca^2+^-free ECS and 2 mM Ca^2+^-containing ECS (140 mM NaCl, 5 mM KCl, 1 mM MgCl_2_, 2 mM CaCl_2_, 10 mM glucose, and 10 mM HEPES buffer; pH adjusted to pH 7.4 with NaOH) for 1 min each. After exposure to 0 mM Ca^2+^ ECS for 2 min, 2 mM Ca^2+^ ECS was re-applied for a further minute. Perfusion continued with 2 mM Ca^2+^ + DMSO (0.2 vol%) for 1 min as internal solvent control and then with either 20 µM pal-PIP_2_ or the control peptide in 2 mM Ca^2+^ ECS or with 0.5 µM cis-22a followed by 20 µM pal-PIP_2_ peptide or vice versa. In any instance, each concentration was applied for 2 min. To altogether prevent Ca^2+^ influx, the experiments ended with perfusion of Ca^2+^-free solution for 2 min. All experiments were repeated on at least three days at room temperature.

Statistical evaluations and blots were carried out using the OriginPro software. F_340nm_/F_385nm_ data were further normalized to the respective minimal value on a per-cell basis for a better comparability, in analogy to [62].

### 4.6. R.Geco1.2 Based Ca^2+^ Imaging

Coverslips carrying HEK293 cells co-expressing YFP-tagged TRPV6 WT or mutant constructs, mCerulean3-CRY2-5′ptase OCRL WT or OCRL D523G, CIBN-CAAX and R.Geco1.2 were washed thrice with 0 mM Ca^2+^ ECS and mounted on the same setup as used for Fura-2 measurements and all fluorescence-based translocation studies. During all preparative steps, pre-activation of CRY2-CIBN was mitigated by keeping cells in darkness and by checking for expression of YFP- or mCerulean3-tagged constructs after the actual Ca^2+^ imaging experiment. Excitation of R.Geco1.2 was achieved using a 500–600 nm LED in combination with a Chroma filter enabling excitation from 540 nm to 580 nm as well as allowing emitted light from 590 to 660 nm to pass, respectively. R.Geco1.2 emission intensity was initially monitored in nominally Ca^2+^-free ECS with a fixed exposure time of 100 ms and a time interval of 10 s between successive iterations. After 1 min, the bath solution was exchanged to provide 2 mM Ca^2+^ in the extracellular space, in analogy to Fura-2 measurements. Following another minute of recording, Ca^2+^ imaging was paused to trigger CRY2-CIBN interactions upon blue light illumination (BLI) with 475 nm for 30 s (maximal lamp output: ~525 mW) with a CHROMA 69,008× ET ECFP/EYFP/mCherry 25mm Dia Mounted filter. Monitoring of R.Geco1.2 emission intensity was consequently continued for a further 3 min under the same conditions as before BLI. After time courses of R.Geco1.2 emission were completed, the same section of the glass slide was illuminated with 425 nm and 475 nm LEDs in combination with a CHROMA 69,008× ET ECFP/EYFP/mCherry 25mm Dia Mounted filter, respectively, to also verify the expression of the phosphatase and TRPV6. Only cells showing fluorescence signals in all cases were marked as regions of interest and R.Geco1.2 emission intensity was further evaluated. To differentiate between reductions in emission intensity caused by the interruption of the measurement necessary for BLI and changes in channel activity due to PIP_2_ depletion, each TRPV6 construct was transfected twice per measurement day, once together with OCRL WT and once with the catalytically inactive OCRL D523G mutant and subjected to the same measurement procedure. Emission values were normalized to the respective minimum and blotted over time using the OriginPro software, which was also implemented for statistical analysis. Therefore, inhibition upon co-expression of OCRL WT or D523G was calculated by dividing the R.Geco1.2 emission intensities determined immediately after BLI by the values before BLI, respectively. Subsequently, the mean inhibition of the OCRL D523G experiments was determined for each co-expressed TRPV6 construct and this value was consequently subtracted from the extents of inhibition calculated for the corresponding OCRL WT-TRPV6 co-expressing cells to derive at Δinhibition.

### 4.7. Subcellular Translocation

To compare cellular localization of the phosphatase system before and after BLI as well as changes in PIP_2_ levels, an mCherry-tagged version of CRY2-5′ptase OCRL and PH-domain were used together with YFP-CIBN-CAAX, whereby expression of the latter was verified just after the translocation study. Accordingly transfected HEK293 cells were washed three times with 2 mM Ca^2+^ ECS and basal localization was recorded for 30 s (three images) in the same solution with the excitation and filter settings as described for R.Geco1.2 measurements. Thereafter, cells were exposed to 475 nm for 30 s to stimulate CRY2 to interact with CIBN and mCherry localization was monitored for a further 90 s with records every ten seconds, respectively.

### 4.8. NFAT Screen

For NFAT translocation studies, HEK293 cells co-expressing TagRFP-hTRPV6 WT with CFP-NFAT were incubated with either 0.1, 1, 5, or 10 μM cis-22a in DMEM + supplements or in the same medium containing the equivalent concentration of DMSO for 2 h at 37 °C. To monitor time dependence of translocation, incubation occurred with 5 μM cis-22a (DMSO) for 1, 2, 3, or 4 h, respectively, or with 10 μM pal-PIP_2_ peptide, the palmitoylated control peptide, or DMSO for 15–20 min or 2 h. TRPV6 mutants were screened for changes in inhibition by cis-22a upon incubation with 5 μM cis-22a or the corresponding dose of DMSO as solvent control. After incubation, cover slips were carefully rinsed with phenol red-free DMEM supplemented with the compound and dosage corresponding to the previous incubation, which was then also present during imaging. In any case, an untreated control was included as well, using phenol red-free DMEM as imaging solution. Subcellular NFAT localization of TRPV6 expressing cells was again monitored on an Axiovert 135 inverted microscope (Zeiss, Oberkochen, Germany), as described in more detail before. Excitation of CFP relied on the 425 nm LED in combination with a CHROMA 69,008× ET ECFP/EYFP/mCherry 25mm Dia Mounted filter. Images were analyzed using Matlab2018a, differentiating between three cell populations based on the ratio of CFP emission intensity originating from the nucleus and the cytosol, similar to Schober et al. (2019) [65].

### 4.9. Statistics

Tests for normal distribution and for the presence of possible outliers were performed before evaluating statistical significance between TRPV6 WT and the individual mutants or different treatments with either unpaired two-sided Student’s *t*-tests, if assuming a normal distribution, or with Mann–Whitney U tests, if a normal distribution was not satisfied. Within bar graphs (mean ± SEM), statistical significance (*p* < 0.05) is indicated by ‘*’. For all electrophysiological measurements and calcium imaging experiments, *n* numbers represent the number of cells contributing to mean values.

## 5. Conclusions

Inhibition of TRPV6 currents by the small molecule blocker cis-22a and PIP_2_ depletion depends on residues within LBS-2 and the pore, whereof most of them are relevant in both cases. The reduced sensitivity of LBS-2 and of most yet not all pore single mutants to inhibition by cis-22a and PIP_2_ sequestration or depletion points to some bidirectional allosteric coupling between LBS-2 and the pore. Thereby, the present findings might contribute to the development of new pharmacological agents inhibiting activity of the oncochannel TRPV6 by mimicking the effects of PIP_2_ depletion.

## Figures and Tables

**Table 1:** Peptide synthesis and purification.

Code	Peptide Sequence	M_mo_ (Calculated) *	M_mo_ (Measured) ^ß^	Rt (min) ^×^
**Pal-PIP_2_ peptide**	**Palmitoyl-HRQKHFEKRR**	1659.0209	1659.0198	16.6
**Control peptide**	**Palmitoyl-HAQKHFEAAA**	1346.7711	1346.7700	18.2

* Theoretical monoisotopic molecular mass. ^ß^ Monoisotopic molecular mass, measured with a Thermo Scientific (Waltham, MA, USA) QExactiv Focus Hybrid Quadrupole-Orbitrap Mass Spectrometer. The deviation of the measured mass from the theoretical mass of the peptides (ΔM) was always lower than 4 ppm. ^×^ Retention time on analytical HPLC chromatograms, using 5–100 B% in 20 min gradient elution on a Phenomenex Jupiter Proteo C12 column (10 μm, 90 Å, 4.6 mm × 150 mm).

## Data Availability

This study includes no data deposited in external repositories. Original data related to this paper will be provided upon reasonable request by the corresponding author.

## Supplementary Materials

The supporting information can be downloaded at: https://www.mdpi.com/article/10.3390/ijms25010618/s1.
