# Supplementary material for: Bidirectional Allosteric Coupling between PIP2 Binding and the Pore of the Oncochannel TRPV6
Source: Int J Mol Sci. 2024 Jan 3;25(1):618. doi: 10.3390/ijms25010618 (PMC10779433; doi:10.3390/ijms25010618)
Supplement: Supplementary file 1 [file ijms-25-00618-s001.zip › Figure S3.pdf]

A

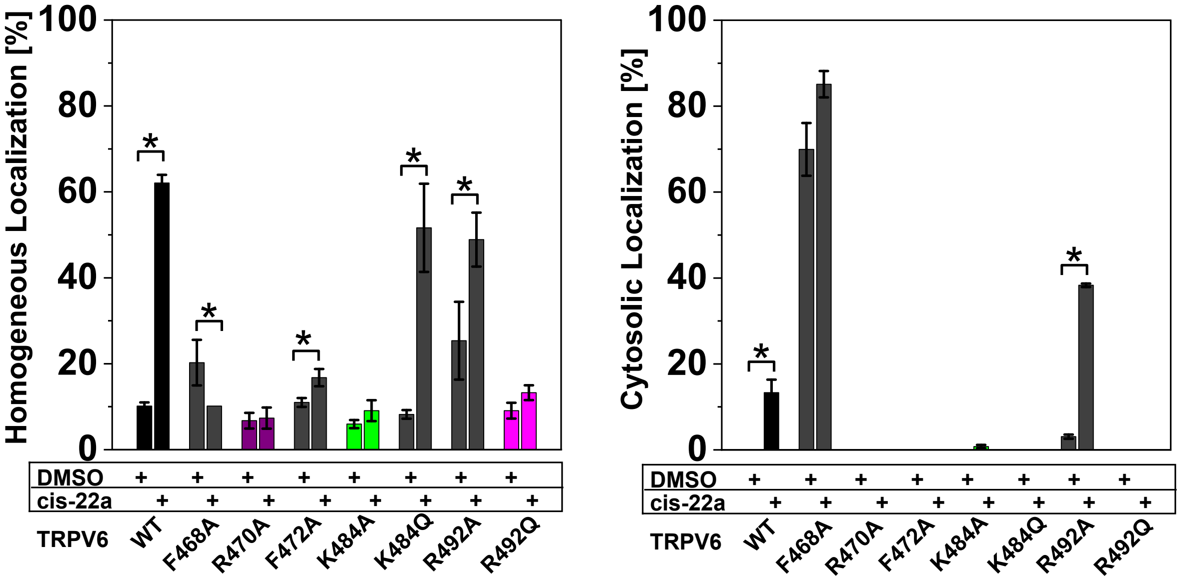

TRPV6	n_{DMSO}	$n_{cis\text{-}22a}$
WT	588	610
F468A	148	148
R470A	283	341
F472A	181	206
K484A	230	225
K484Q	180	198
R492A	103	109
R492Q	83	70

B

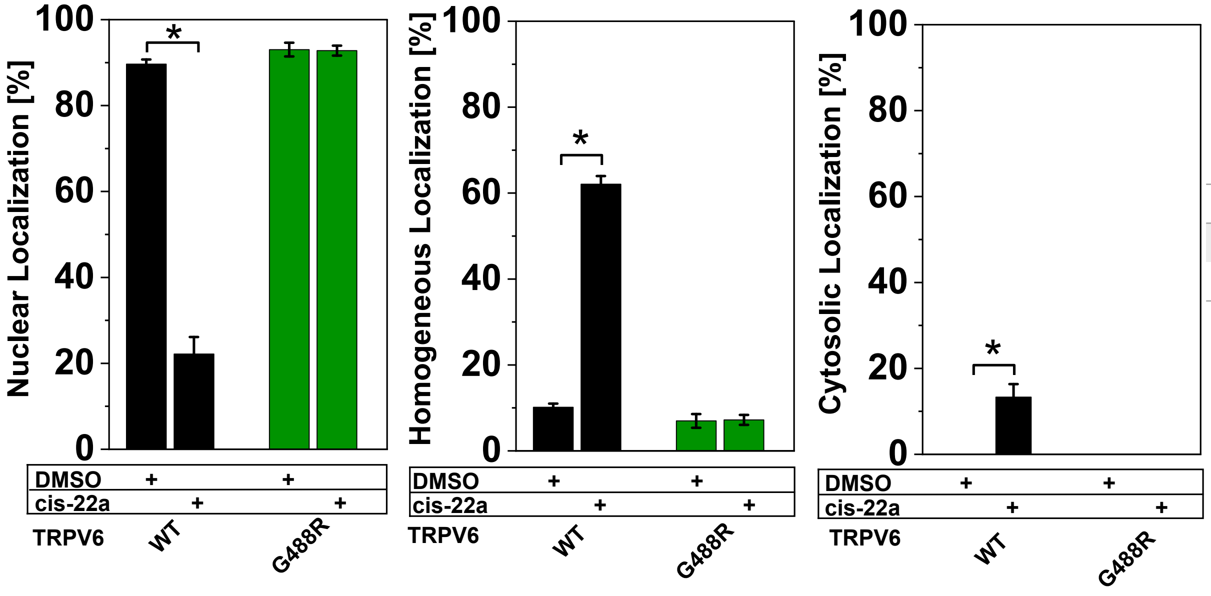

TRPV6	n_{DMSO}	$n_{cis\text{-}22a}$
WT	274	203
G488R	73	70

Supplementary Figure S3
