# Supplementary material for: Bidirectional Allosteric Coupling between PIP2 Binding and the Pore of the Oncochannel TRPV6
Source: Int J Mol Sci. 2024 Jan 3;25(1):618. doi: 10.3390/ijms25010618 (PMC10779433; doi:10.3390/ijms25010618)
Supplement: Supplementary file 1 [file ijms-25-00618-s001.zip › Supplementary Figure Legends.pdf]

Figure S1: Dose-dependent inhibition of TRPV6 by PIP_2 sequestration. (A) Interface between three TRPV6 subunits (A green, B yellow, D light blue) depicting the four different lipid binding sites (LBS-1-4) as purple meshes. The subunit in front was removed for clarity. **(B)** Left: average (mean – SEM) whole-cell current densities recorded from HEK293 cells expressing TRPV6 WT normalized to the maximum. Measurements were recorded in 10 mM Ca^{2+} solution that was consecutively exchanged by the same solution containing DMSO, 10 µM pal-PIP_2 peptide (black solid line) or the control peptide (grey dashed line), followed by La^{3+}, as indicated. Right: Corresponding extents of inhibition in %. **(C)** Normalized whole-cell measurements (mean – SEM) corresponding to **(B)** derived upon doubling (20 µM) the concentration of the pal-PIP_2 peptide and of the control peptide. **(D)** Exemplary current-voltage relationship of HEK293 cells expressing TRPV6 WT upon applying a voltage ramp from -90 mV to + 90 mV within 200 ms in the presence and absence of 10 µM pal-PIP_2 peptide. **(E)** Averaged whole-cell current densities (- SEM) of HEK293 cells transfected with TRPV6 WT (black) or TRPV6 G488R (green). Cells were successively perfused with 10 µM pal-PIP_2 peptide and 1 mM $La^{3+,}$ as indicated by the bars (left), and with the resultant mean percentages of inhibition by 10 µM pal-PIP_2 peptide relative to La^{3+} (right). Asterisks (*) indicate statistical significance ($p<0.05$) between different treatments **(B, C)** or between TRPV6 WT and TRPV6 G488R **(D)**.

Figure S2: TRPV6 activity-dependent localization pattern of NFAT (A) Representative fluorescence images of untreated HEK293 cells co-expressing CFP-tagged NFAT and Tag-RFP-labelled TRPV6. **(B)** Average (mean ± SEM) localization pattern (nuclear, homogeneous, cytosolic) of HEK293 cells co-transfected with CFP-NFAT and TagRFP-TRPV6 in the absence of a specific treatment. **(C)** Mean (± SEM) percentage of cells showing NFAT in the nucleus (black), the cytosol (light grey) or an indifferent, homogeneous distribution (dark grey) after incubation with 5 µM DMSO for different durations (1-4 h), serving as control for the measurements in **(D)** upon incubation with 5 µM cis-22a for the same incubation times. **(E)** Localization (mean ± SEM) pattern of NFAT in TRPV6-expressing HEK293 cells following incubation with 10 µM pal-PIP_2 peptide, the palmitoylated control peptide (control) or the corresponding dose of DMSO for 15-20 minutes or **(F)** for two hours.

Figure S3: NFAT localization of TRPV6 pore mutants after treatment with cis-22a. (A) Mean (± SEM) percentage of cells expressing the indicated LBS2-mutant and NFAT showing a homogeneous (left) or a cytosolic (right) localization of the transcription factor after incubation with 5 µM DMSO or cis-22a for 2 h and the corresponding total number of cells (right) involved in the analysis. **(B)** Cellular distribution (nuclear: left, homogeneous: middle, cytosolic: right bar chart) of NFAT after treating CFP-NFAT and TagRFP-TRPV6 WT or G488R expressing HEK293 cells for two hours with DMSO as control or with 5 µM cis-22a; the panel on the far right summarizes the total cell numbers implicated in the analysis. In all charts, asterisks (*) indicate statistically significant differences between both treatments ($p<0.05$).

Figure S4: Validation of optogenetic PIP_2 depletion using a PIP_2 sensor and the TRPV6 G488R mutant as functional control. (A) Exemplary fluorescence records showing localization of a sensor for PIP_2, a mCherry-tagged PH-domain, in HEK293 cells in the resting state (left) and after blue light illumination (BLI) (right) induced interactions between mCerulean3-CRY2-5´ptase OCRL WT and YFP-CIBN containing a C-terminal CAAX box to enable membrane anchorage. **(B)** Localization of the N-terminally mCherry-tagged CRY2-5´ptase OCRL WT construct upon rest (left) and after exposure to 475 nm for 30 s (right) as well as localization of co-expressed YFP-CIBN-CAAX. For **(A)** and **(B)**, the scale bar approximates 10 µm. **(C)** Ca^{2+} imaging data on optogenetic PIP_2 depletion in HEK293 cells upon co-expressing TRPV6 WT (left) or TRPV6 G488R (middle) with prenylated CIBN, mCerulean3-tagged CRY2-5´ptase OCRL WT or the D523G mutant, respectively. The difference in inhibition in the presence of OCRL WT and OCRL D523G derived from the time traces on the left (normalized mean R.Geco1.2 emission ± SEM) was used to evaluate TRPV6 WT or TRPV6 G488R inhibition by phosphatase activation (right, mean ± SEM). The asterisk (*) indicates a statistically significant difference in the response to PIP_2 depletion between TRPV6 WT and the mutant ($p<0.05$).

Figure S5: NFAT localization in the presence of TRPV6 pore and C-terminal mutants after treatment with cis-22a. (A) Mean (± SEM) percentage of HEK293 cells co-expressing TRPV6 WT or the mentioned pore mutant and NFAT that show a homogeneous (left) or a cytosolic (right bar chart) localization of the transcription factor following treatment with 5 µM DMSO or cis-22a for two hours and the corresponding total cell numbers whereon evaluations were based (right). **(B)** Proportion of HEK293 cells (mean ± SEM) depicting a nuclear (left), homogeneous (middle) or cytosolic (right) localization of fluorescently labeled NFAT upon co-expression with TRPV6 C-terminal mutants and after incubation with DMSO or cis-22a (5 µM) for two hours and the corresponding total number of cells (lower panel). **(C)** Exemplary fluorescence images showing localization patterns of CFP-tagged NFAT and TagRFP-labeled TRPV6 R589A in HEK293 cells after incubation for two hours with either DMSO or 5 µM cis-22a. The scale bar approximates 10 µM. For all bar charts, asterisks (*) indicate statistically significant differences in the respective cellular localization between both treatments ($p<0.05$).

Figure S6: Response of TRPV6 R470A W583F to cis-22a and washout experiments. (A) Cellular distribution of NFAT after treating CFP-NFAT and TagRFP-TRPV6 WT or TRPV6 R470A W583F expressing HEK293 cells for two hours with DMSO as control or with 5 µM cis-22a; the lower panel summarizes the total cell numbers implicated in the analysis of the localization patterns. The asterisks (*) indicate statistically significant differences between both treatments ($p<0.05$). **(B)** Left: Mean (-SEM) whole-cell current density of TRPV6 WT or TRPV6 R470A W583F expressing HEK293 cells recorded in 10 mM Ca^{2+} solution which was successively supplemented with DMSO, 0.1 µM cis-22a, 10 µM cis-22a or 1 mM La^{3+}, as indicated by the bars. Right: Corresponding mean (± SEM) levels of inhibition reached by 0.1 µM and 10 µM cis-22a relative to the La^{3+} block. The asterisks (*) show statistical significance between TRPV6 WT and the double mutant ($p<0.05$). **(C)** Average (mean – SEM) whole-cell current densities of HEK293 cells expressing TRPV6 WT recorded in 10 mM Ca^{2+} solution which was consecutively exchanged by the same basal solution containing either 0.1 µM cis-22a, 10 µM DMSO or 1 mM La^{3+}. **(D)** Average (mean – SEM) whole-cell current densities of HEK293 cells expressing TRPV6 WT recorded in 10 mM Ca^{2+} solution which was consecutively exchanged by the same basal solution containing either 10 µM pal-PIP_2 peptide, 0.1 µM DMSO or 1 mM La^{3+}.

Figure S7: TRPV6 structure. (A) Schematic on the domain organization of the TRPV6 protein (NH: N-terminal helix, ARD: ankyrin repeat domain, β1/2/3: beta strand 1/2/3, LH1/2: linker helix 1/2, pre-TM1: pre- transmembrane domain 1 helix, TM1-6: transmembrane domain 1-6, P: pore helix). **(B)** Cryo-EM structure of human TRPV6 in the open (left, PDB: 7K4A) and closed (right, PDB: 6BOA) state with the residues R470 (purple), K484 (green), R492 (magenta), I575 (cyan), D580 (blue) and W583 (red) shown as sticks. For a better visualization, the subunits in the front and back have been removed in **(B)** and **(C):** cryo-EM structure of human TRPV6 (PBD: 7K4B) in complex with cis-22a (orange) and zoom-in to the pore binding site depicting the cubic cage formed by the TRPV6 W583 residues with the individual channel subunits highlighted in different colors.
