# Supplementary material for: Bidirectional Allosteric Coupling between PIP2 Binding and the Pore of the Oncochannel TRPV6
Source: Int J Mol Sci. 2024 Jan 3;25(1):618. doi: 10.3390/ijms25010618 (PMC10779433; doi:10.3390/ijms25010618)

Pal-PIP$_2$ Mmo(meas)=1659.0198

HK_016 #28-41 RT: 0.12-0.18 AV: 14 NL: 4.37E8
T: FTMS + c ESI Full ms [100.0000-1500.0000]

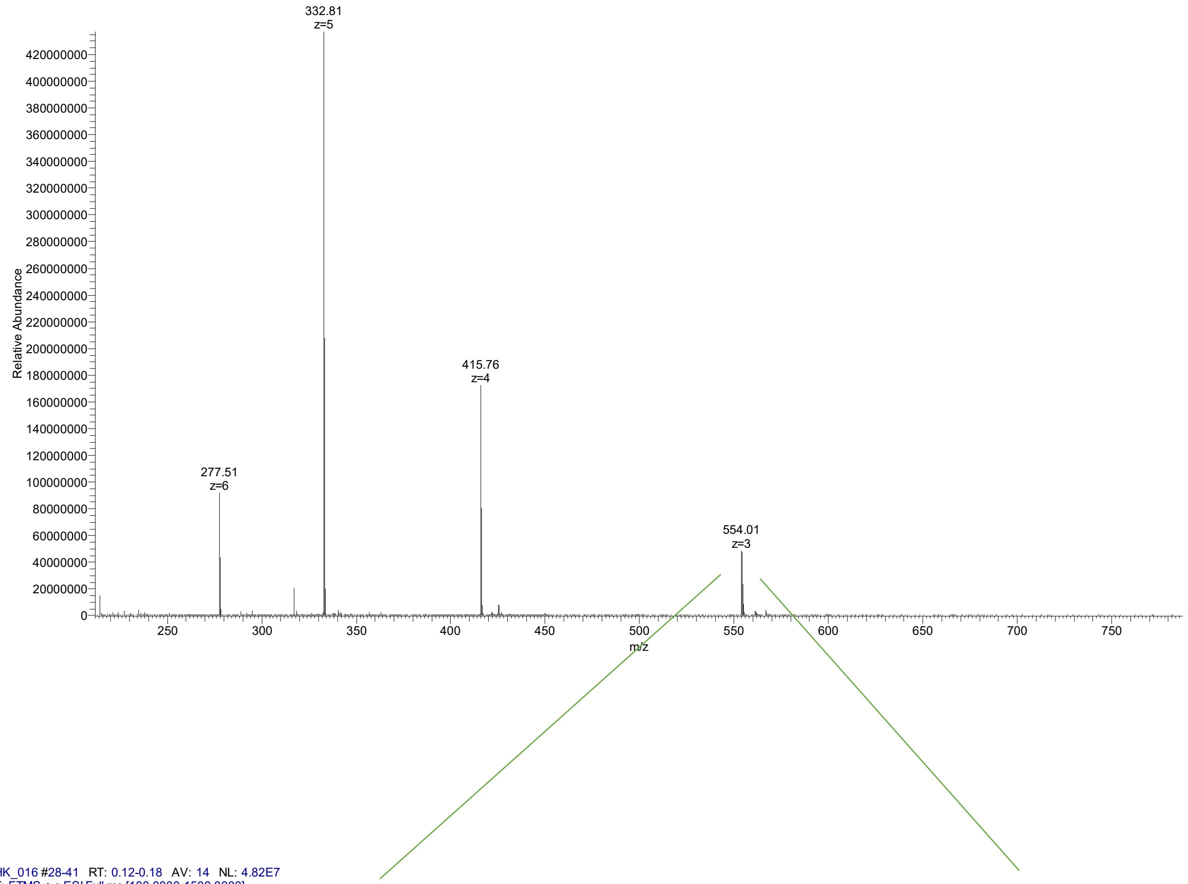

HK_016 #28-41 RT: 0.12-0.18 AV: 14 NL: 4.82E7
T: FTMS + c ESI Full ms [100.0000-1500.0000]

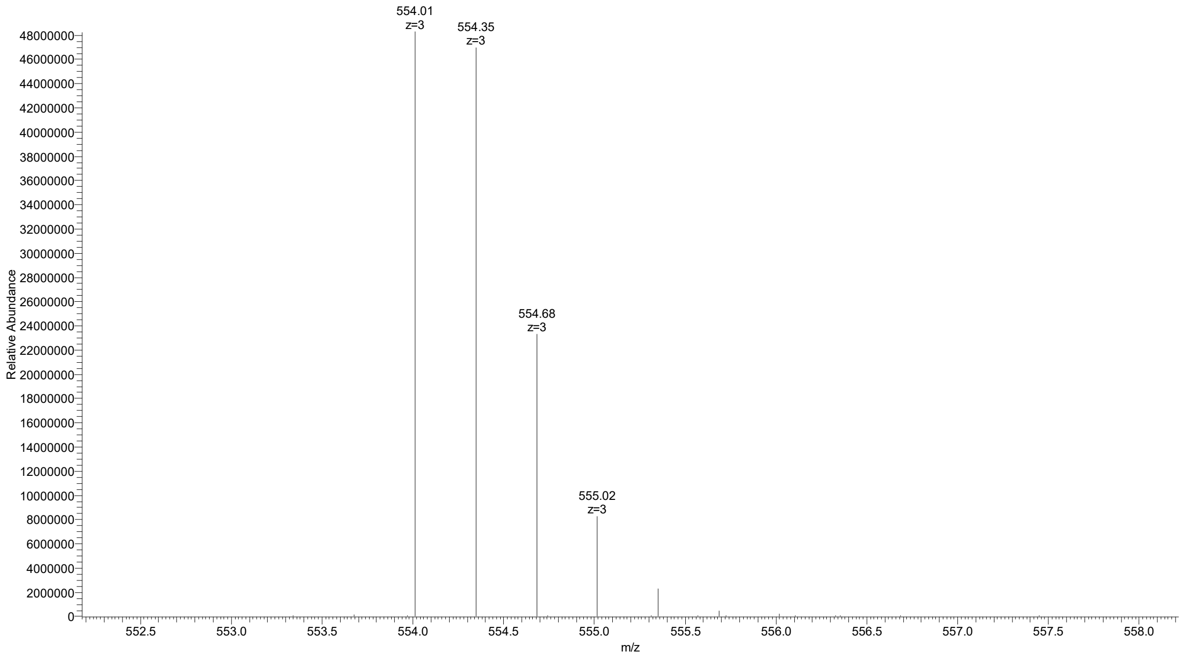

Pal-AA-PIP$_2$ Mmo(meas)=1346.7700 (and its Na+ adduct)

hk_017 #27-41 RT: 0.12-0.18 AV: 15 NL: 3.19E8
T: FTMS + c ESI Full ms [100.0000-1500.0000]

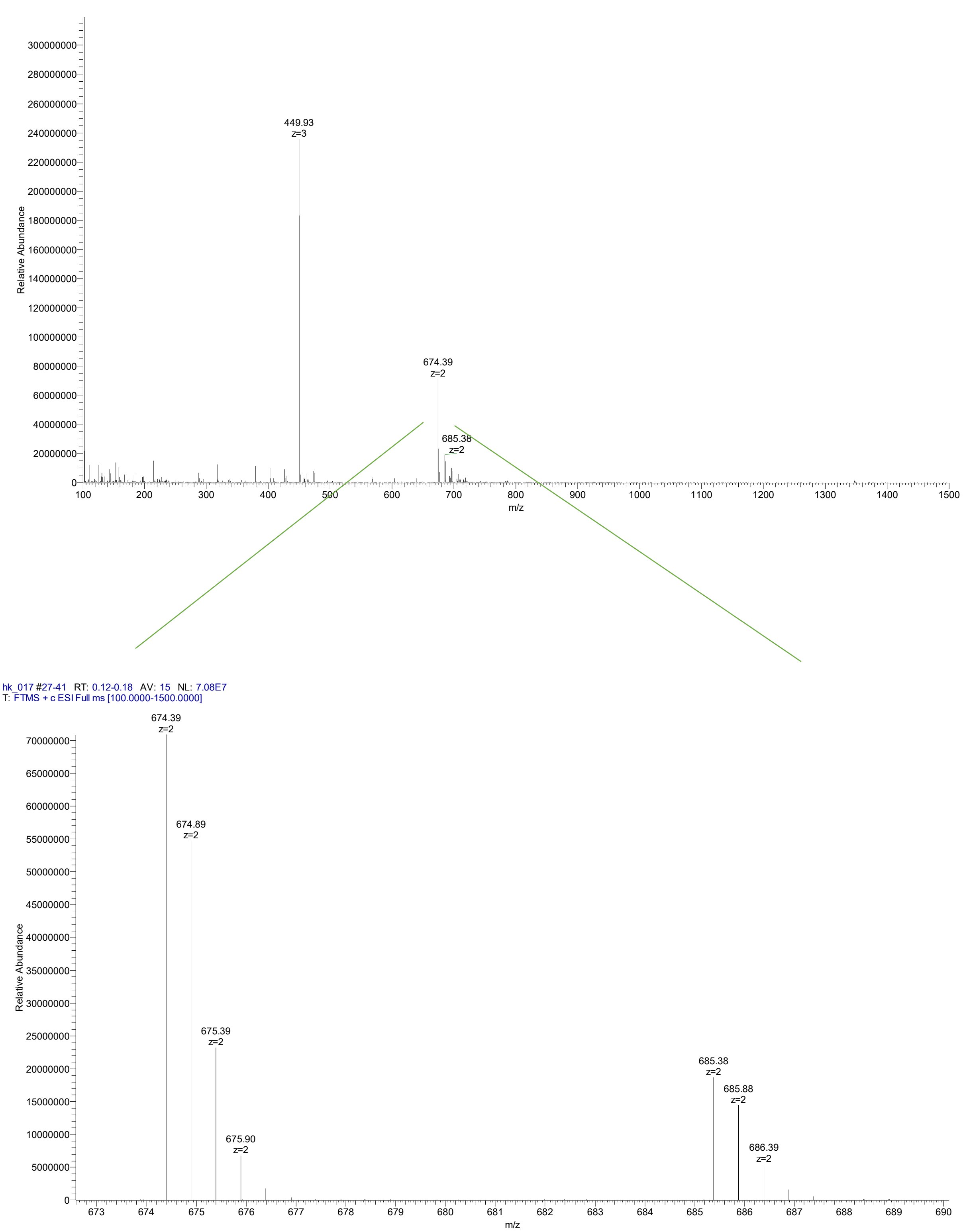

Supplement: Supplementary file 1 [file ijms-25-00618-s001.zip › Supplementary Information.pdf]
