# Supplementary figures and images for: Bidirectional Allosteric Coupling between PIP2 Binding and the Pore of the Oncochannel TRPV6
Source: Int J Mol Sci. 2024 Jan 3;25(1):618. doi: 10.3390/ijms25010618 (PMC10779433; doi:10.3390/ijms25010618)

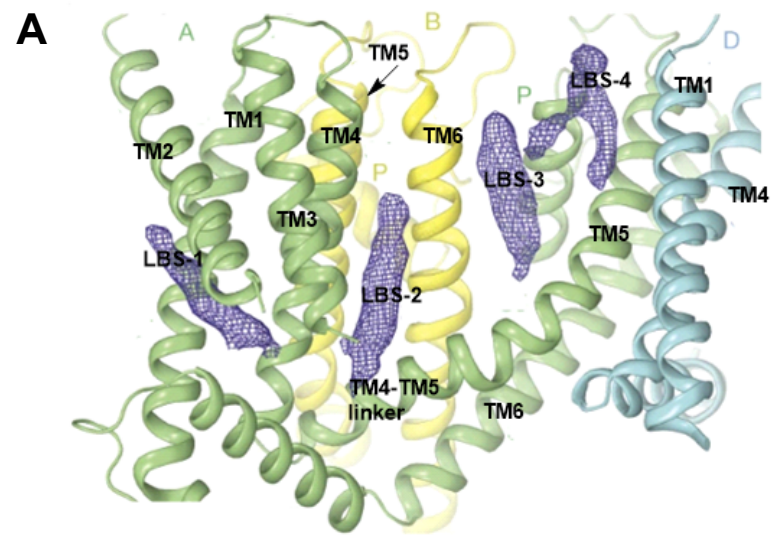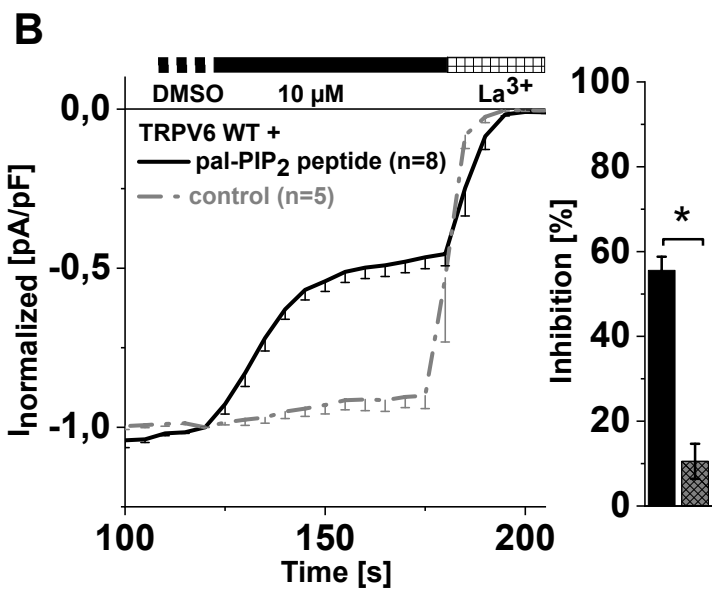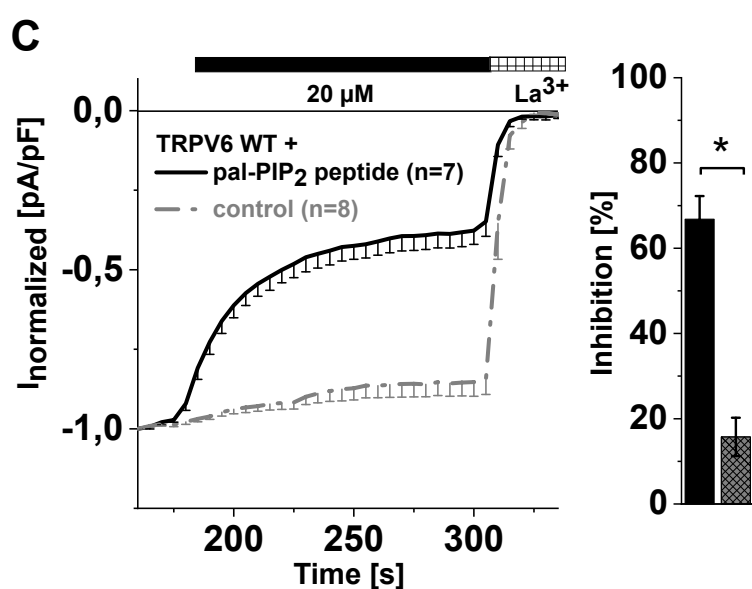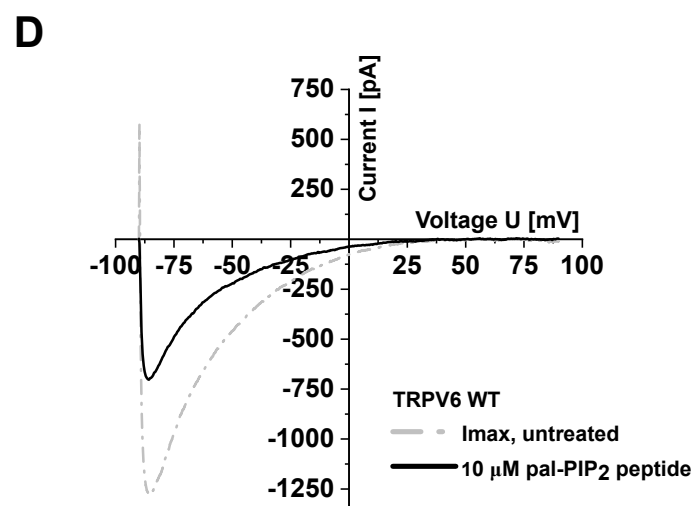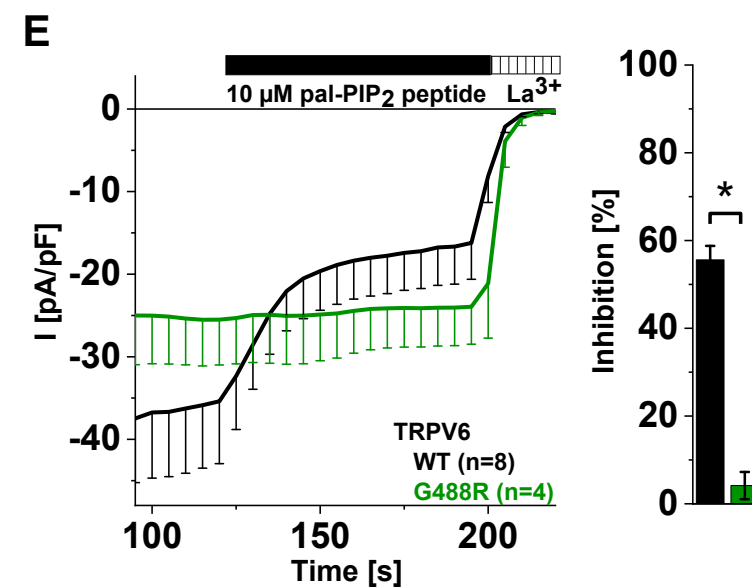

Supplementary Figure S1

Supplement: Supplementary file 1 [file ijms-25-00618-s001.zip › Figure S1.pdf]

**A**

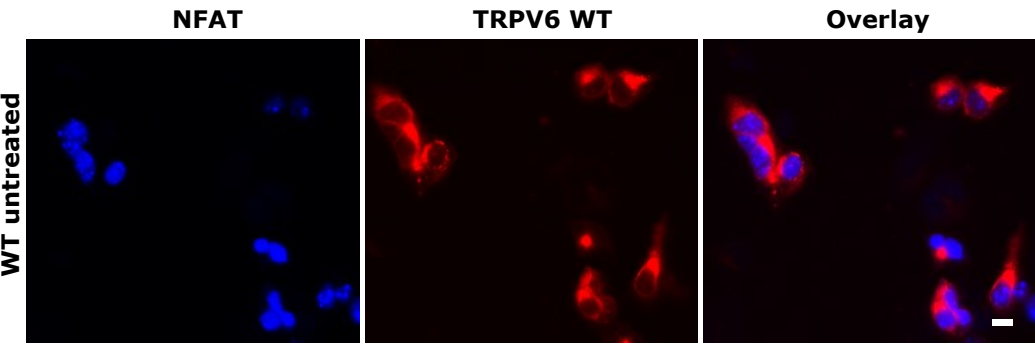

**B**

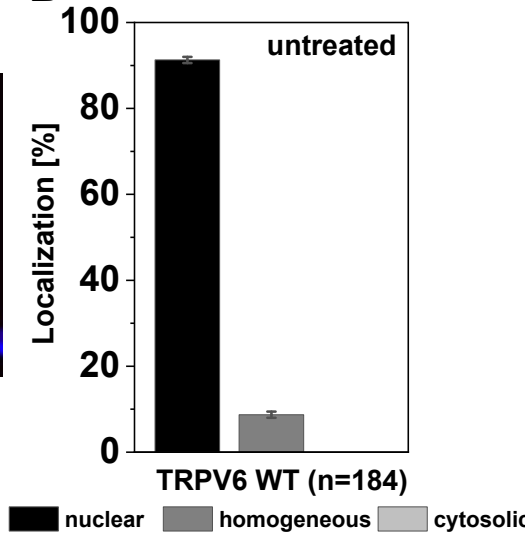

**C**

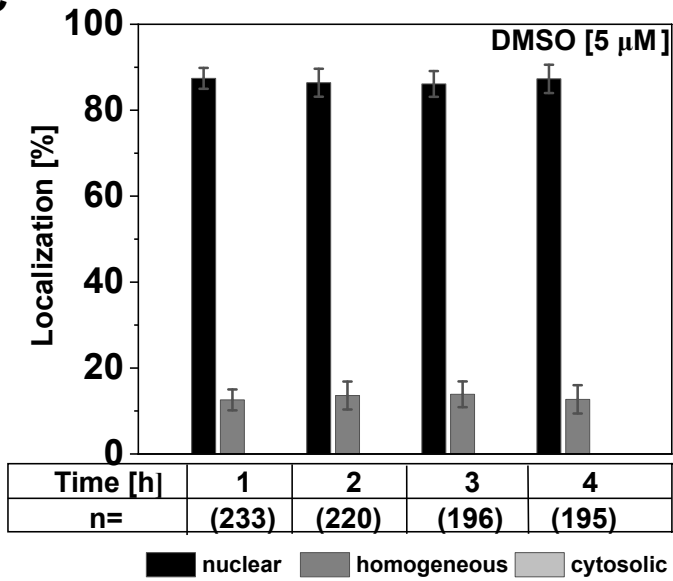

**D**

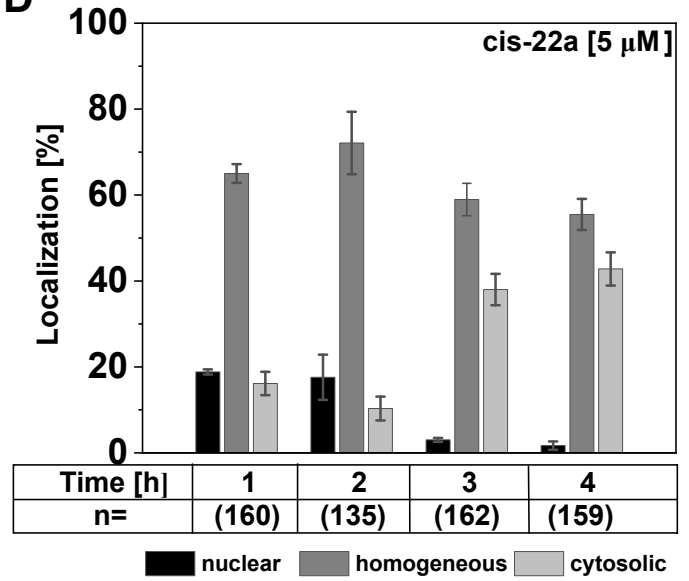

**E**

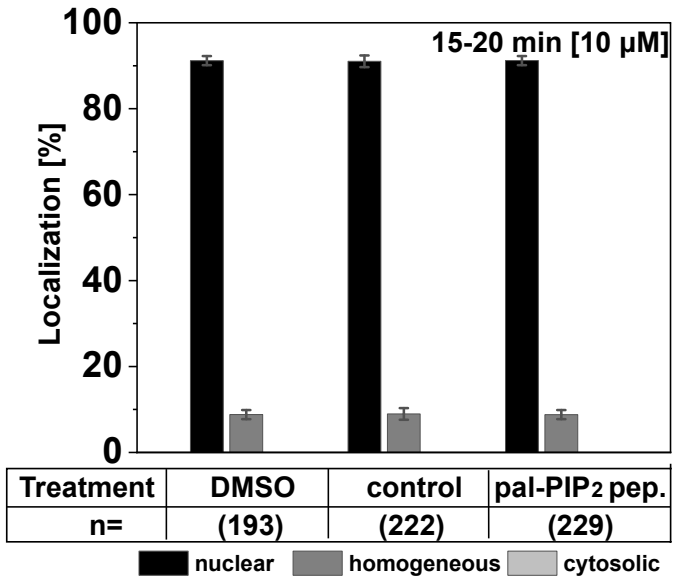

**F**

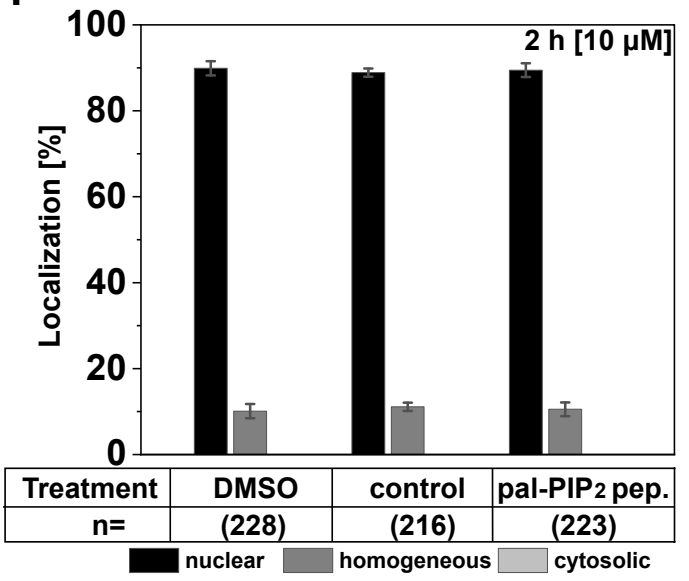

Supplement: Supplementary file 1 [file ijms-25-00618-s001.zip › Figure S2.pdf]

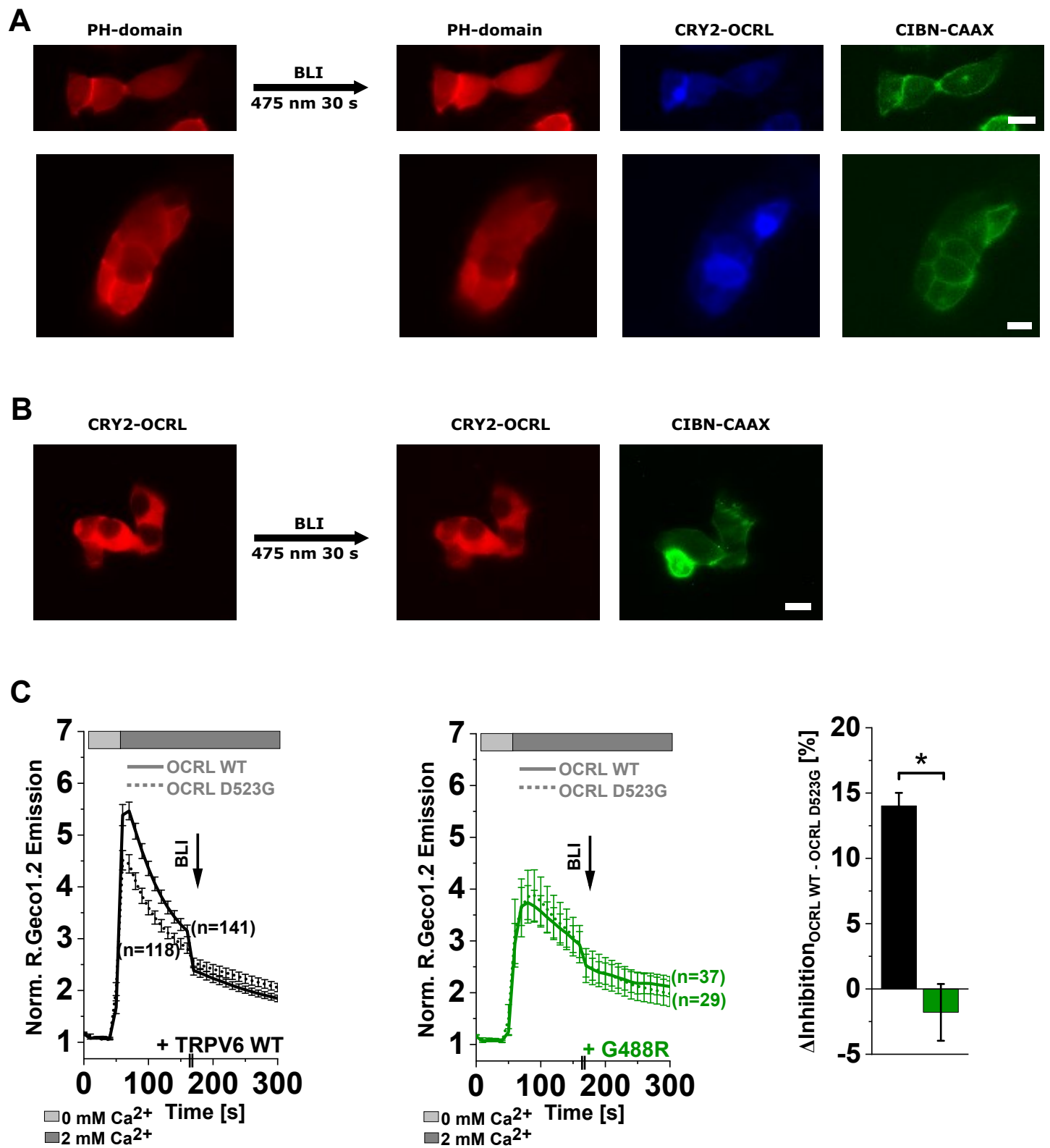

Supplementary Figure S4

Supplement: Supplementary file 1 [file ijms-25-00618-s001.zip › Figure S4.pdf]

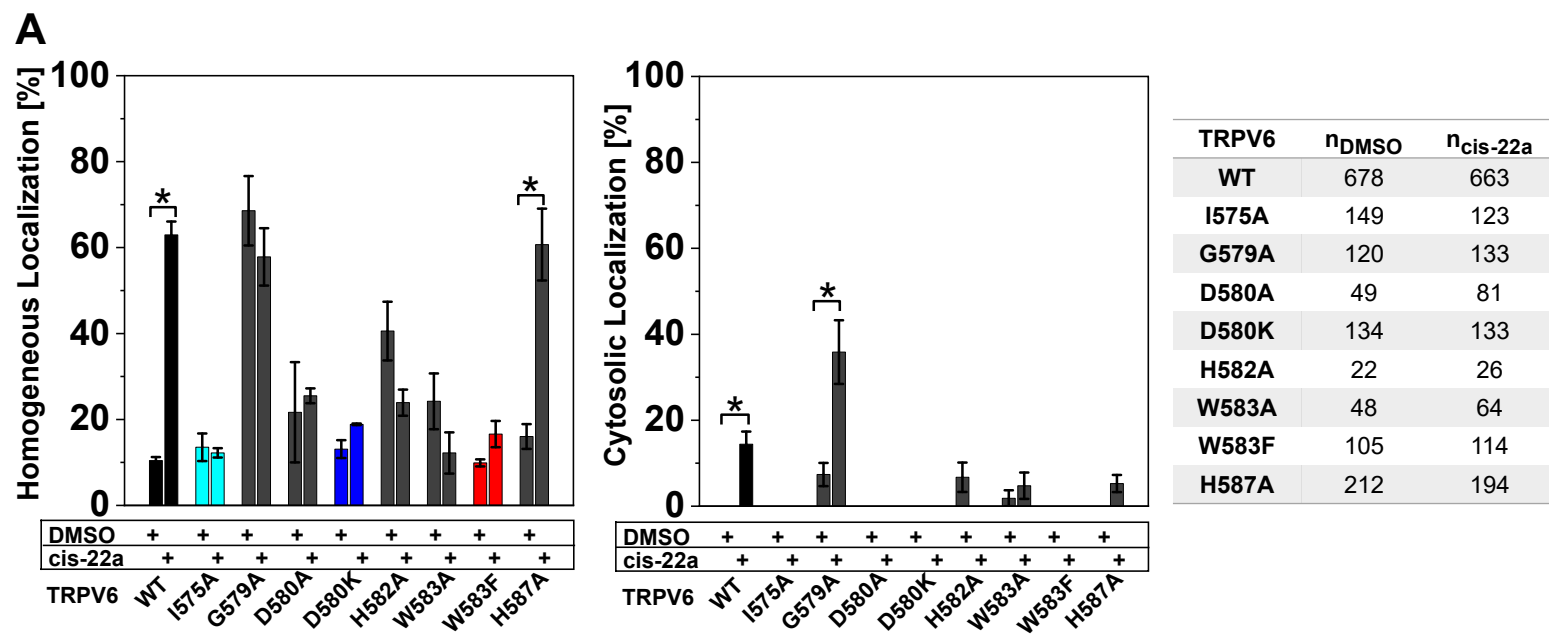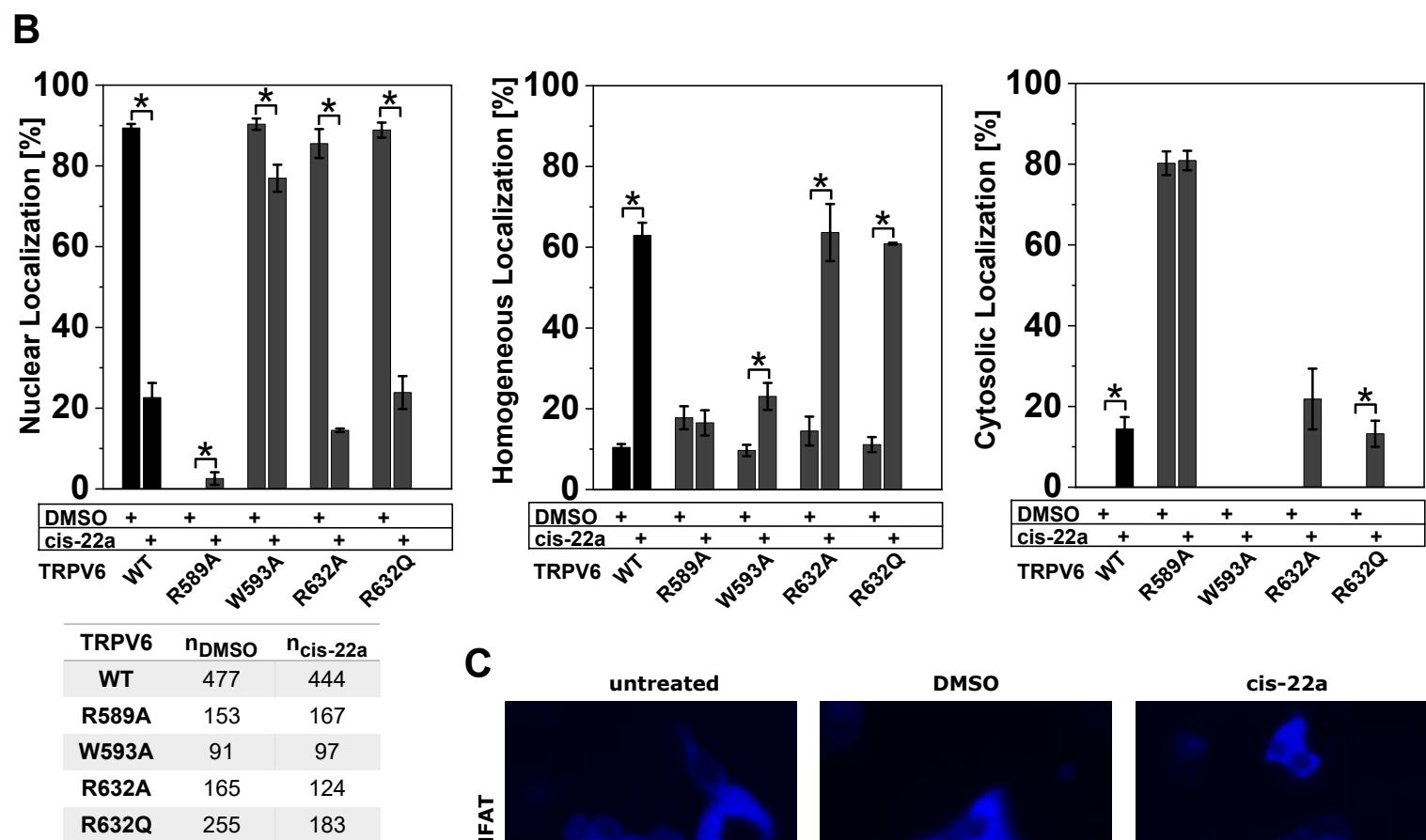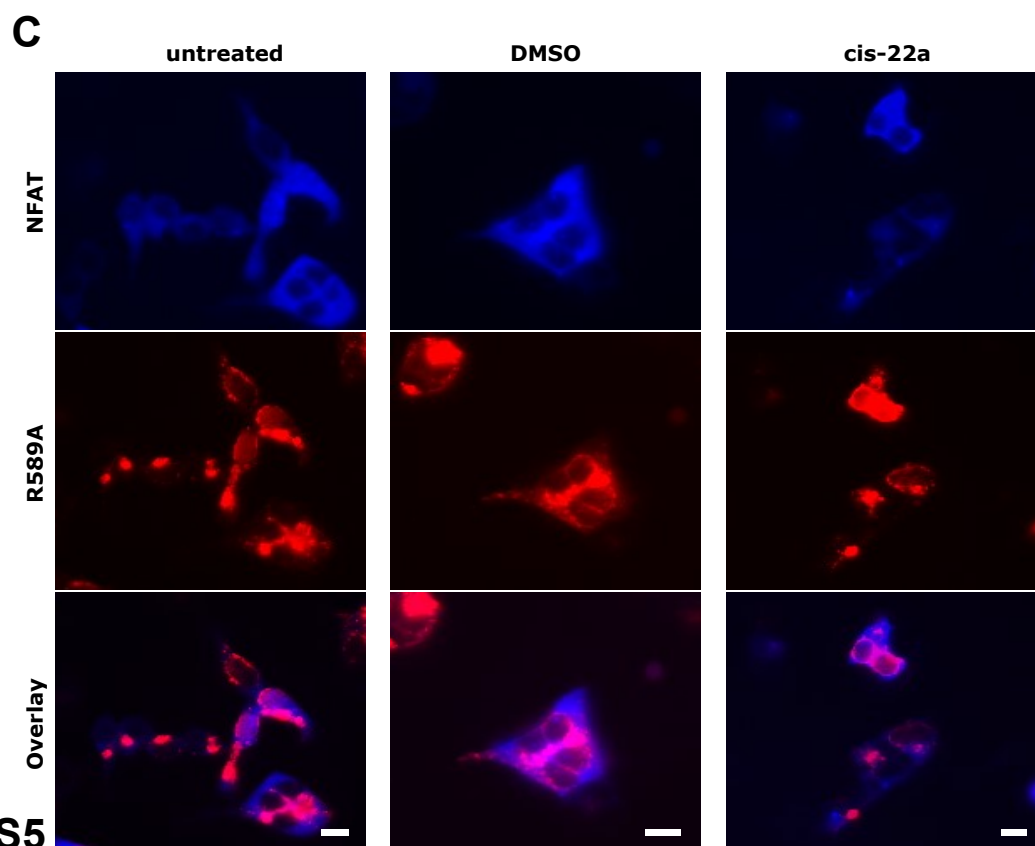

Supplementary Figure S5

Supplement: Supplementary file 1 [file ijms-25-00618-s001.zip › Figure S5.pdf]

**A**

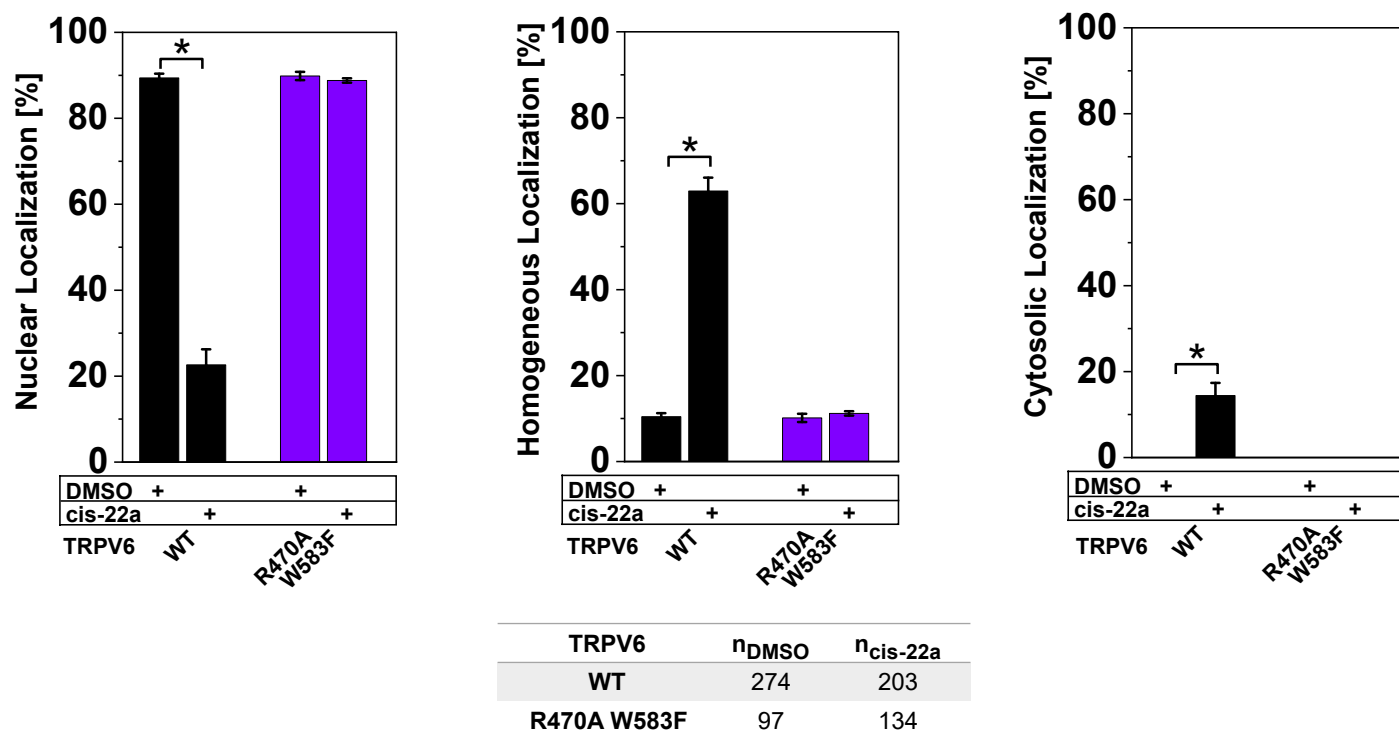

**B**

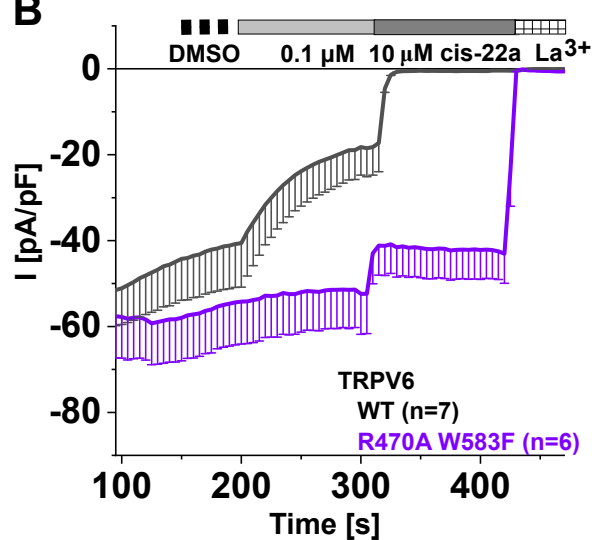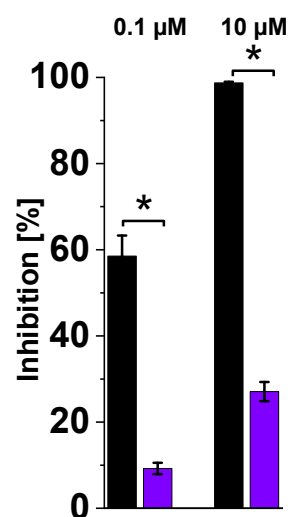

**C**

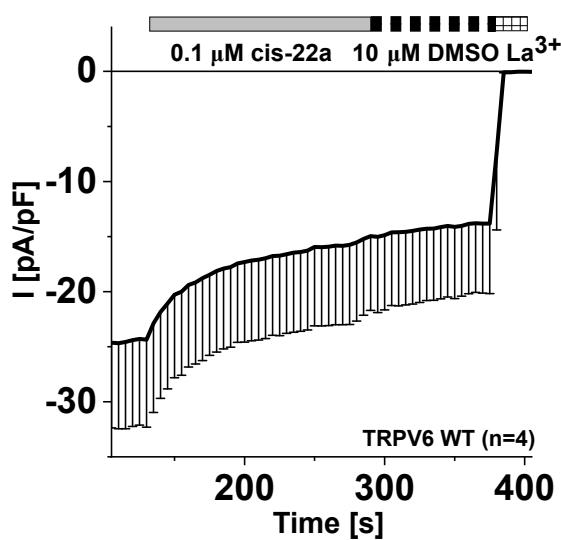

**D**

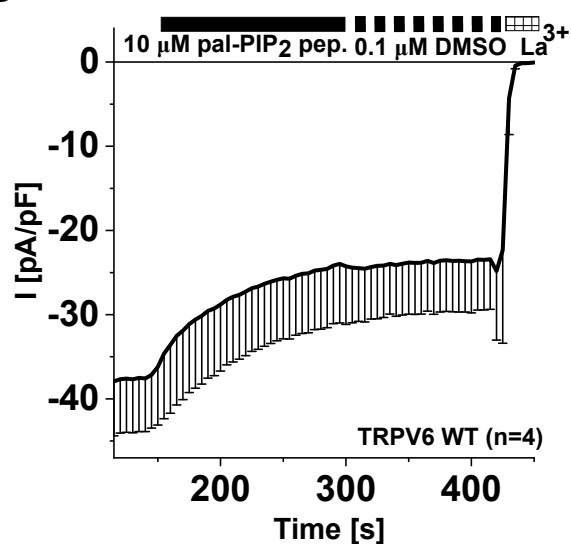

Supplementary Figure S6

Supplement: Supplementary file 1 [file ijms-25-00618-s001.zip › Figure S6.pdf]

A

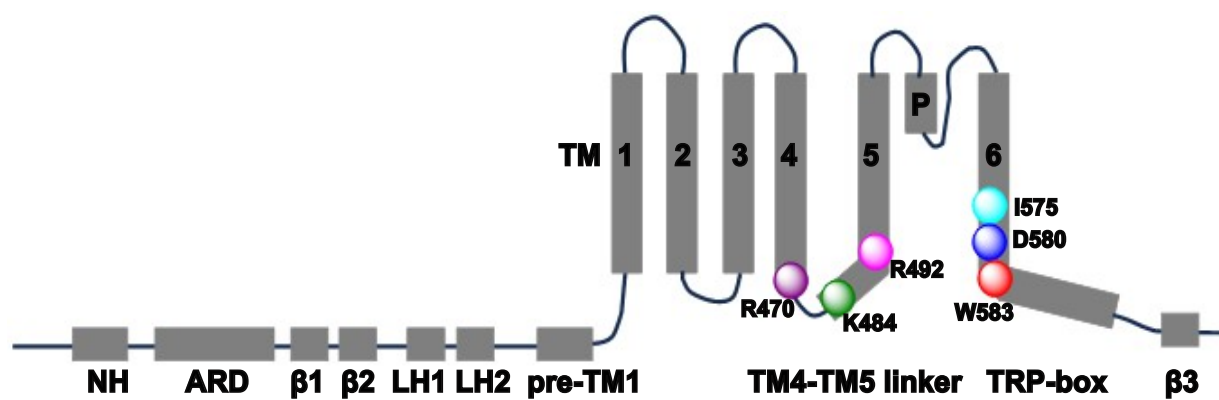

B

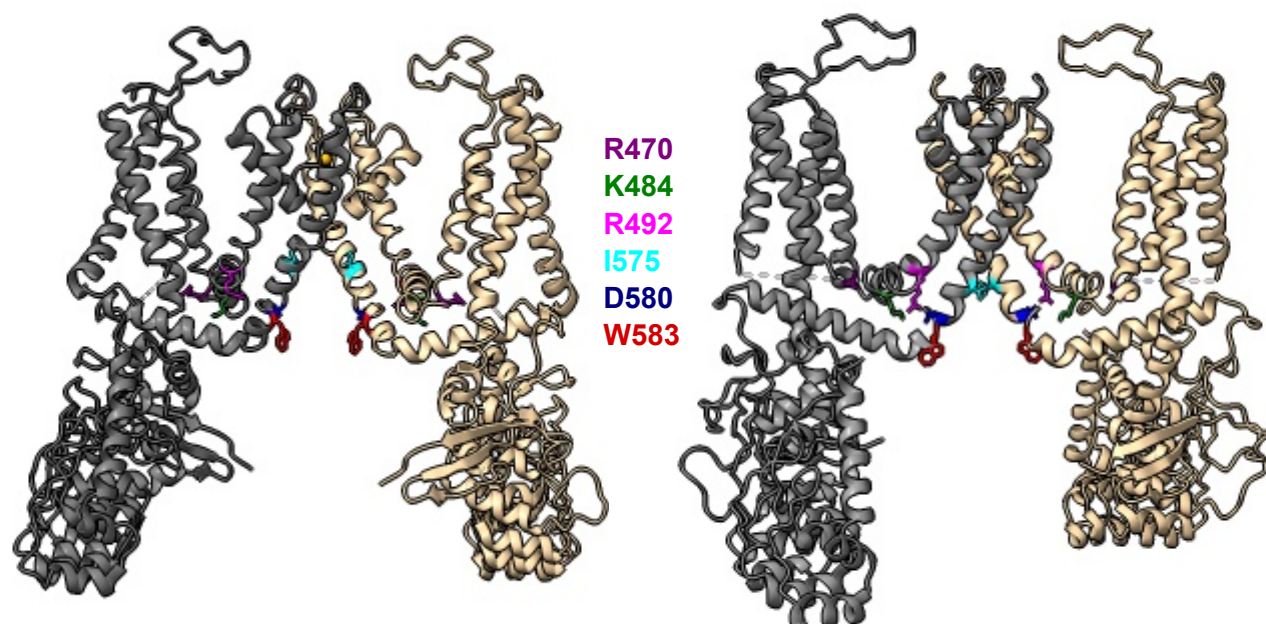

C

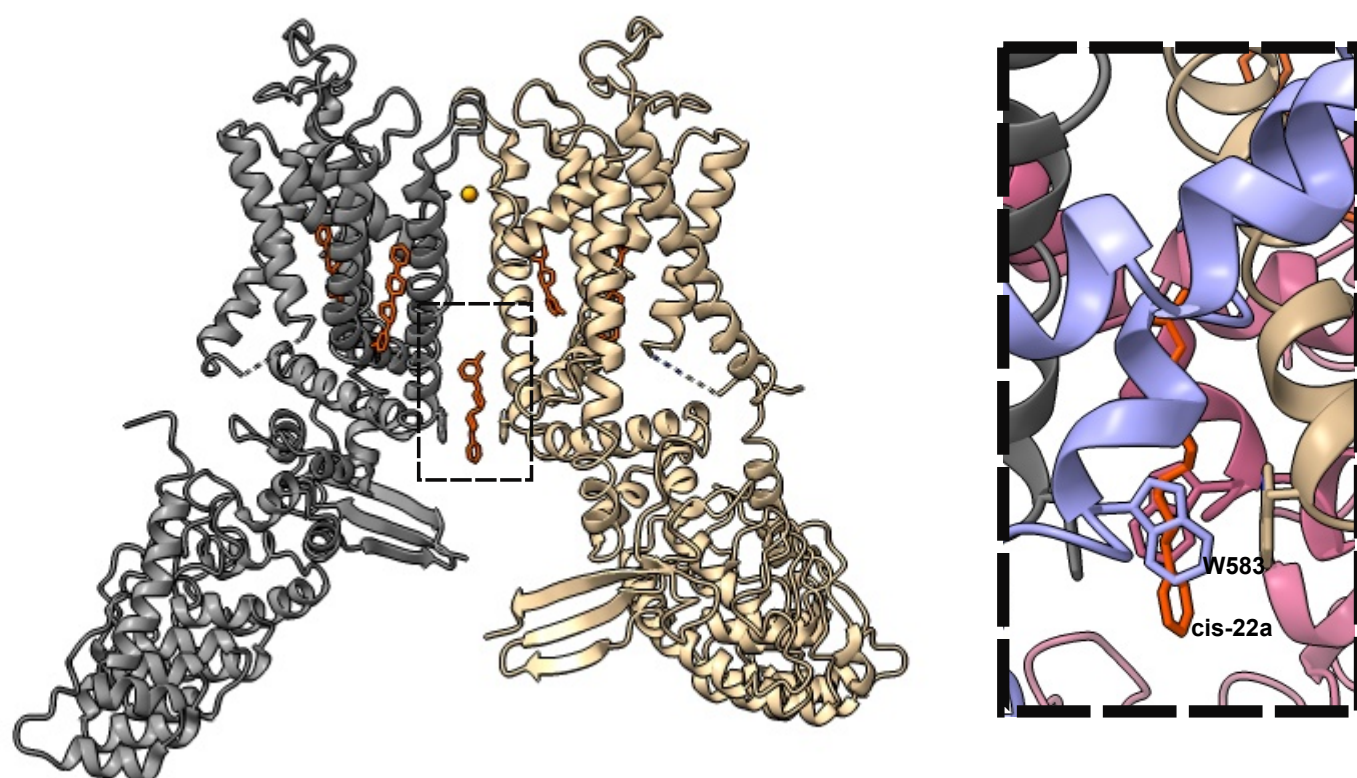

Supplementary Figure S7

Supplement: Supplementary file 1 [file ijms-25-00618-s001.zip › Figure S7.pdf]
